# To click or not to click for short pulse-labeling of the bacterial cell wall†

**DOI:** 10.1039/d4ra04945d

**Published:** 2024-10-21

**Authors:** Morgane Baudoin, Anne Chouquet, Mai Nguyen, André Zapun, Basile Pérès, Cécile Morlot, Claire Durmort, Yung-Sing Wong

**Affiliations:** a Univ. Grenoble Alpes, CNRS, DPM 38000 Grenoble France yung-sing.wong@univ-grenoble-alpes.fr; b Univ. Grenoble Alpes, CNRS, CEA, IBS 38000 Grenoble France claire.durmort@ibs.fr

## Abstract

A method of choice to study the spatio-temporal dynamics of bacterial cell growth and division is to analyze the localization of cell wall synthesis regions by fluorescence microscopy. For this, nascent cell wall biopolymers need to be labeled with fluorescent reporters, like fluorescent d-alanines (FDAs) that can be incorporated into the peptidoglycan. To achieve high spatial and temporal resolution, dense, high-intensity fluorescence labeling must be obtained in the shortest possible time. However, modifications carried by d-Ala can hinder their uptake by the enzymes that incorporate them into the peptidoglycan, such as the d,d-transpeptidases. Conversely, these modifications can impede the elimination of the incorporated d-Ala derivatives by d,d-carboxypeptidases, making the labeling more persistent. In this context, we synthesized clickable d-Alas and tested their incorporation into the peptidoglycan using different labeling approaches, prior or after their conjugation to clickable fluorescent dyes through SPAAC reaction. Our data allow ranking of the d-Ala derivatives in terms of their ease of incorporation and resistance to trimming during one-step, “one-pot” two-step or sequential two-step labeling strategies. We further show that a hybrid “one-step” approach, in which a FDA is used in combination with clickable choline and fluorescent dye, enables two-color co-labeling of peptidoglycan and teichoic acids. Finally, we identify a strategy compatible with the cell fixation required for super-resolution microscopy, by combining one-step labeling with FDA and sequential two-step labeling with clickable choline and fluorescent dye, allowing to obtain two-color high-resolution images of peptidoglycan and teichoic acid synthesis regions.

## Abstract 2

Clicking fluorescent dyes to live bacteria at the right time enables high-intensity fluorescence, and high spatial and temporal resolution.

## Graphical abstract

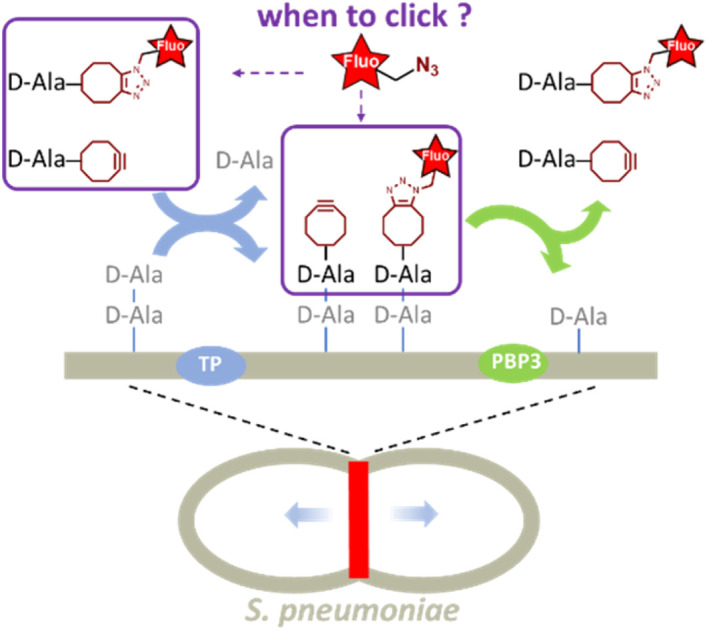

## Introduction

The bacterial cell wall is an essential structure that plays a crucial role in maintaining the shape and the structural integrity of the cell. In Gram-positive bacteria, it is composed of polymeric layers of peptidoglycan (PG), which is the main and ubiquitous component in the bacterial kingdom, and teichoic acids (TAs), which are polyphosphosaccharidic chains covalently linked to the PG or to membrane lipids. Their co-assembly during cell division and elongation is dynamically controlled by the cell wall synthesis machinery, making this step a prime therapeutic target with major challenges in understanding the underlying mechanisms.^1^

To study and understand the expansion of the cell wall, it is essential to dispose of specific labeling tools that can track the genesis of the PG or the TAs. There are several strategies based on the incorporation of modified metabolites, which are constituents of bacterial wall biopolymers. Among them, sugar derivatives are an effective means of labeling or installing a clickable group in the cell wall.^2,3^ To specifically label the PG, d-alanine (d-Ala) derivatives are commonly used due to their ease of chemical synthesis and selectivity.^2–4^ The metabolic incorporation of clickable d-Ala derivatives into the PG has provided highly accurate monitoring of its fate during bacterial division and elongation.^3,4^ This has been made possible by conjugating the incorporated clickable probes to fluorescent dyes carrying complementary clickable functions, and subsequent localization using super-resolution microscopy.^5,6^

**Fig. 1:**
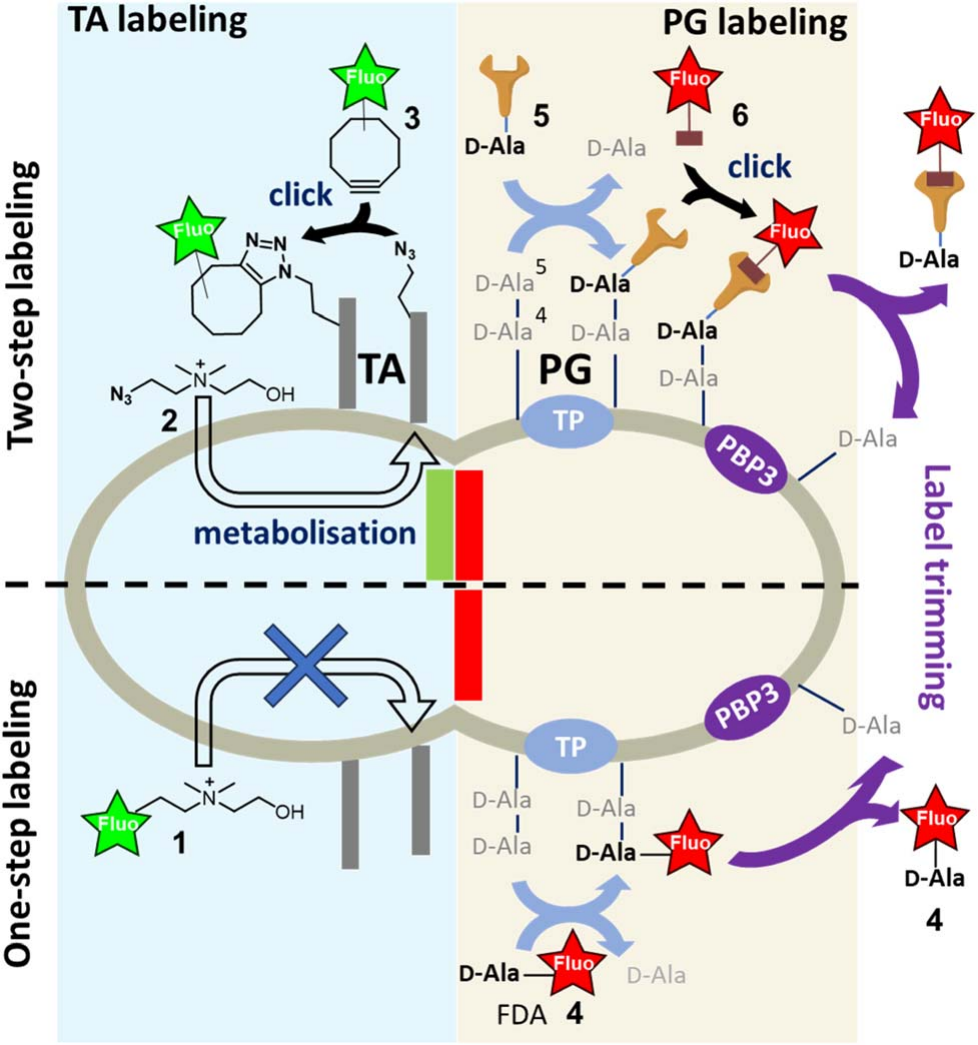
Fluorescent labeling of nascent cell wall components by one- or two-step labeling approach; teichoic acids (TA) are labeled by the metabolic incorporation of azido-choline 2 followed by SPAAC reaction with fluorescent cyclo-octyne 3 (two-step labeling); peptidoglycan (PG) can be labeled either by one-step labeling with the incorporation of a fluorescent d-alanine (FDA) 4 or by two-step labeling with clickable d-alanine (d-Ala) 5 that reacts with the matching clickable fluorescent dye 6. Trimming by PBP3 of the terminal d-Ala, carrying either the clickable function or the fluorescent dye, is indicated by purple arrows.

For TAs, we developed their first selective fluorescent labeling by taking advantage of their decoration by phosphocholine groups, which is a particularity of *Streptococcus pneumoniae* and enables specific metabolic labeling using modified choline.^7^ It should be noted that this metabolic incorporation has limited steric tolerance, as the presence of a fluorescent coumarin tag on choline 1 (Fig. 1) does not result in labeling. A two-step labeling approach was therefore set up, involving the metabolic incorporation of azido-choline 2 (azido-Cho), which positions several azide functions in the TAs. The latter can then react with a fluorescent cyclo-octyne 3 by strain-promoted azide–alkyne click reaction (SPAAC).^8^ Given that the metabolism of choline is rapid, we developed a “one-pot” two-step approach, which consists of adding azido-Cho 2 and the fluorescent dye 3 at the same time, thereby increasing the temporal resolution of TA labeling.^8^ In this approach, the key to success lies in the rapid metabolic incorporation of the azido-Cho 2 into the TAs, combined with grafting of the fluorescent dyes by click reaction.

The PG is constituted by polysaccharide strands made of *N*-acetylglucosamine (GlcNAc)-*N*-acetylmuramic acid (MurNAc) repeating units, the latter bearing a pentapeptide that harbors a d-alanine-d-alanine (d-Ala-d-Ala) sequence at its distal C-terminus. The incorporation of newly synthesized glycan strands into the PG mesh requires the cross-linking of the peptide chains. In *S. pneumoniae*, this process is carried out by d,d-transpeptidases (TPs), which remove the terminal d-alanine (d-Ala) to establish a covalent link between the d-Ala in position 4 and an amine-bearing side chain of another peptide. Alternatively, when a significant amount of free d-Ala is present in the medium, TPs can replace the departing d-Ala in position 5 by another d-Ala. This enzymatic futile cycle allows to incorporate a fluorescent d-Ala (FDA) at this stage, enabling the detection of TP activity and serving as a proxy for PG synthesis. However, this one-step incorporation can be impaired by the size of the fluorescent dye,^9,10^ making the approach unsuitable for short pulse-labeling, which needs to be fast and intense. The alternative is thus to use a two-step approach by introducing the fluorescent dye *via* a copper-free click reaction after the metabolization of a d-Ala carrying a small TP-compatible clickable function. SPAAC is one of the most widely used reactions for this purpose. The azide group and the cyclo-octyne are uncharged and sufficiently small to be well tolerated by enzymes. In addition, the SPAAC kinetics are fast enough to usually ensure sufficient grafting of the fluorescent dye and obtain strong labeling in just a few minutes. This strategy also offers the opportunity to use a wide variety of fluorescent probes, particularly those compatible with super-resolution dSTORM (direct STochastic Optical Reconstruction Microscopy), regardless of their size or charge. However, the two-step labeling approach requires more handling because of successive additions and washings, similar to a pulse-chase sequence. In addition, after incorporation of the d-Ala derivatives in living cells, the labeled regions will evolve spatially during the click reaction period and their location will no longer accurately reflect the regions of PG neosynthesis. In such case, the multiple successive steps will decrease the spatio-temporal resolution of the experiment. The transposition of the “one-pot” two-step labeling concept developed for TA to the labeling of PG is therefore highly desirable. It should be noted that PG labeling by modified d-Ala presents a difficulty compared to TA labeling. Indeed, the persistence of the labeling can be quickly compromised by the action of d,d-carboxypeptidases (CPs), which convert the pentapeptides into shorter tetrapeptides by removing the FDA in position 5.^11^ TPs and CPs thus have opposite actions in terms of probe incorporation. Furthermore, the clickable function carried by the d-Ala, whether conjugated or not to a bulky fluorescent group, can greatly influence their uptake by enzymes. In the case of a “one-pot” two-step labeling approach, where all the ingredients are added at the same time, all these parameters increase the complexity in interpreting the efficiency of d-Ala incorporation, fluorescent labeling and maintenance of the incorporated probes.

In this work, the properties of three cyclo-octynes with different reaction kinetics and steric hindrance were tested under various addition schemes to exploit the action of TPs and CPs for strong pulse labeling. Depending on the application, for conventional fluorescence microscopy with live cells or for dSTORM with fixed cells, we sought to identify the optimal operating way for carrying out pulse and pulse-chase experiments with the best temporal resolution. With these conditions in hand, we have extended our work to co-labeling of the PG and TA, and produced the first two-color dSTORM localization images of nascent PG and TA.

## Results and discussion

### Reaction kinetics of d-Ala-cyclo-octynes with azide fluorescent dyes

**Scheme 1:**
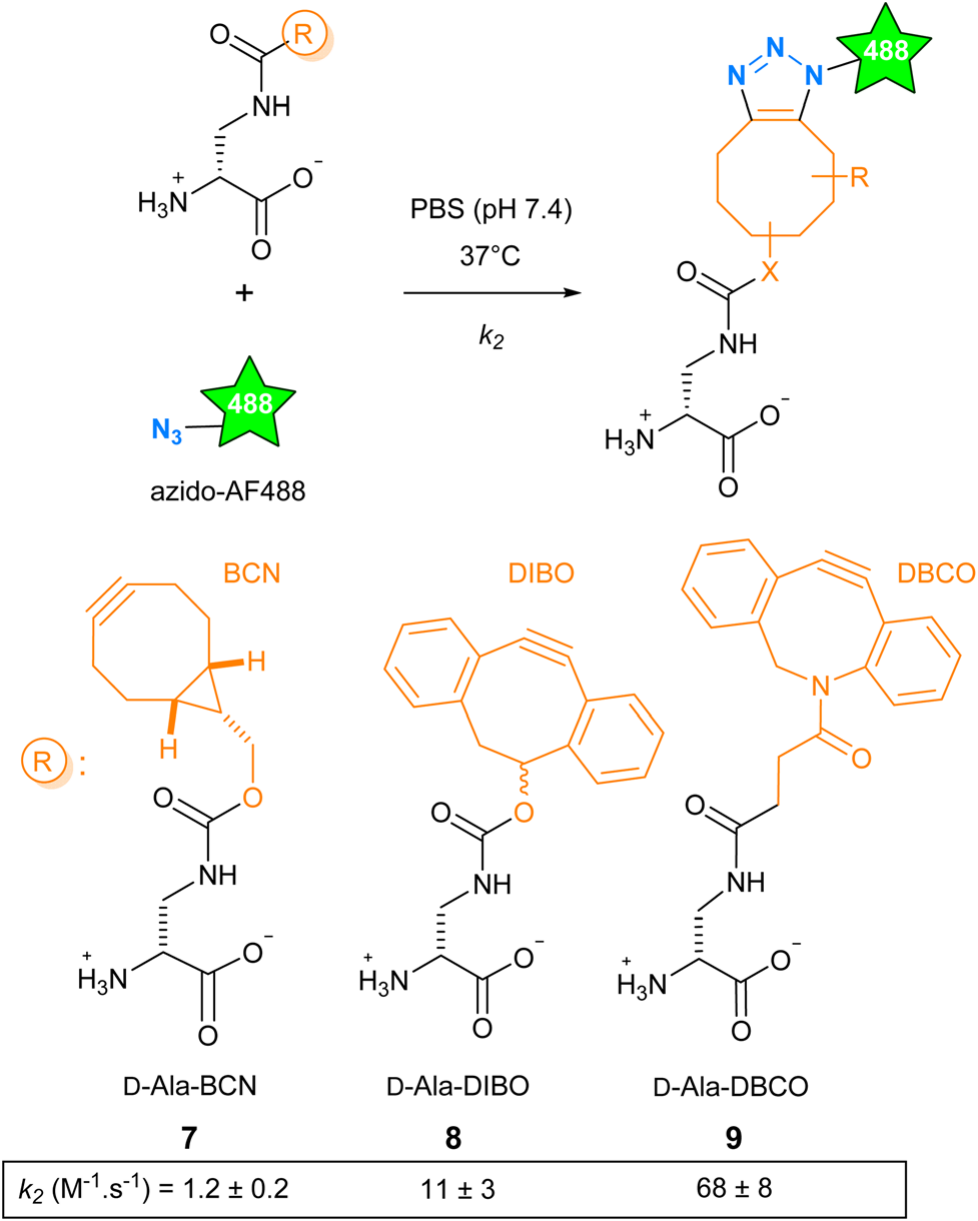
Formation of FDAs through the conjugation of d-Ala-BCN 7, d-Ala-DIBO 8 or d-Ala-DBCO 9 to an azido derivative of the fluorescent dye AF488. The SPAAC reaction kinetics in PBS at 37 °C is given below each compound.

The SPAAC reaction can be carried out with functions of various sizes, from the small azide group to more voluminous polycyclic cyclo-octynes, and offers a range of reaction kinetics. We have explored how these parameters influence cell wall labeling for fluorescence microscopy. We selected the cyclo-octynes BCN, DIBO and DBCO because fluorescent dyes carrying these functions or their matching clickable functions are commercially available (Fig. S1†). The second-order rate constant *k*_2_ reported for BCN (in methanol), DIBO (in CD_3_CN/D_2_O 3 : 1) and DBCO (in methanol) reacting with aliphatic azides at room temperature was reported to be 1.4 × 10^−1^, 1.2 × 10^−1^ and 3.1 × 10^−1^ M^−1^ s^−1^, respectively.^12^ The d-Dap (amino-d-alanine), as a central scaffold mimicking the d-Ala, was chosen to accommodate these cyclo-octynes, giving d-Ala-BCN 7,^13^d-Ala-DIBO 8 and d-Ala-DBCO 9.^14^ We then carried out a comparative study of the reaction kinetics between 7–9 and azido-AF488 in biologically compatible condition (phosphate buffer saline (PBS), 37 °C, Scheme 1). Compared to the literature, we found a ten-fold higher *k*_2_ value for d-Ala-BCN (1.2 ± 0.2 M^−1^ s^−1^) and much higher ones for d-Ala-DIBO (11 ± 3 M^−1^ s^−1^) and d-Ala-DBCO (68 ± 8 M^−1^ s^−1^). This marked difference may be explained by the change in temperature and medium, but also by the presence of the d-Dap moiety carried by the clickable molecule.^15^ As a result, the range of kinetics is wider than anticipated, with excellent kinetics for 8 and 9.

### Comparison of labeling approaches in the study of PG neosynthesis

**Fig. 2:**
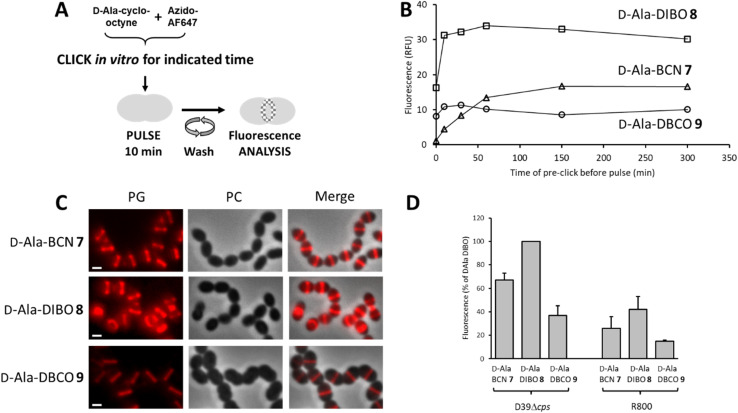
(A) One-step pulse-labeling scheme. *S. pneumoniae* cells are incubated for 10 min with a solution of FDA (d-Ala-(7–9) pre-clicked with azido-AF647). (B) Fluorescence of labeled cells measured by flow cytometry for various time of pre-click reaction between products 7–9 and azido-AF647. The time to reach the labeling intensity plateau indicates the pre-click time required for full conversion to FDA. (C) Conventional fluorescence microscopy images of cells labeled with FDA, following a pre-click reaction between product 7–9 and azido-AF647 over a period of 2.5 h. Left panels show the peptidoglycan (PG) synthesis region revealed by the AF647 signal, middle panels show phase contrast (PC) images and right panels show merged phase contrast and AF647 channels. All images were recorded with the same acquisition parameters, and signal levels were treated identically for each panel. Scale bars, 1 μm. (D) Histogram showing the relative fluorescence intensity of labeled *S. pneumoniae* D39 Δ*cps* and R800 cells analyzed by flow cytometry. The FDAs used for labeling were obtained by pre-click reactions of 2.5 h. The fluorescence intensity measured for D39 Δ*cps* cells labeled with the FDA issued from compound d-Ala-DIBO 8 and azido-AZ647 was taken as a reference.

To label the PG, the one-step labeling approach was first tested (Fig. 2A). A solution of 7–9 was pre-incubated (pre-clicked) for different periods with the fluorescent dye azido-AF647 in PBS buffer to determine the minimum incubation time for full conversion into the corresponding FDA. The reaction products were used to label the PG of the laboratory strain D39 Δ*cps S. pneumoniae*, and the fluorescence of labeled cells was measured by flow cytometry to estimate the relative yield of FDA conversion (Fig. 2B). Reaching a plateau of labeling intensity was considered of the consequence of the full conversion of the d-Ala-cyclo-octynes into FDA during the preincubation. Consistent with the reaction kinetics measured *in vitro*, a plateau was reached after a few tens of minutes of pre-click reaction for 8 and 9, whereas it took 2.5 h for d-Ala-BCN 7. Interestingly, the labeling intensity was not the same between FDAs, with the pre-clicked d-Ala-DIBO 8 and d-Ala-DBCO 9 compounds providing the strongest and weakest signals, respectively. Fluorescence microscopy images of pneumococcal cells labeled with compounds pre-clicked for 2.5 h show midcell localization patterns that are typical of probes incorporated into newly synthesized PG (Fig. 2C). The labeling intensity obtained for the different compounds mirrors that observed with flow cytometry. The variations observed between the different FDAs likely reflect differences in their ability to be incorporated by the TPs and susceptibility to be trimmed by PBP3, the single pneumococcal CP. In the other widely used laboratory strain *S. pneumoniae* R800, d-Ala-DIBO 8 also yielded the strongest fluorescent signal, albeit less intense than in strain D39 Δ*cps* (Fig. 2D). To compare fluorescence intensities in the subsequent experiments, whenever possible, 10 min pulse-labeling of WT strains with d-Ala-DIBO was taken as a reference (normalized reference = 100).

**Fig. 3:**
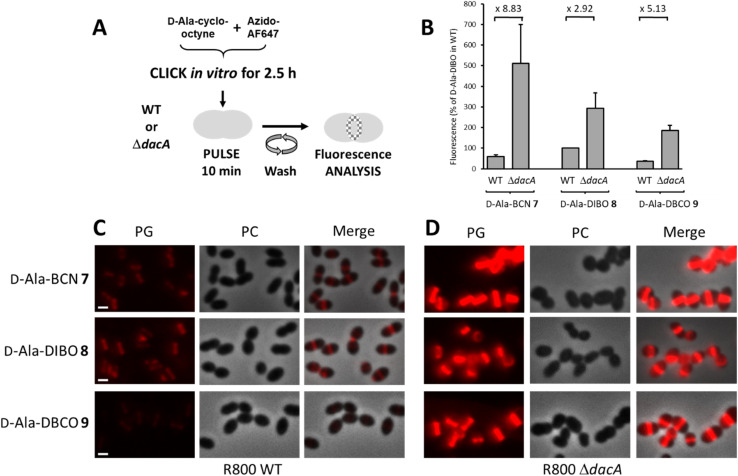
(A) One-step pulse-labeling scheme. *S. pneumoniae* R800 WT and Δ*dacA* cells are incubated for 10 min with a solution of FDA (d-Ala (7–9) pre-clicked with the azido-AF647 for 2.5 h). (B) Histogram showing the relative fluorescence intensity of labeled R800 WT and Δ*dacA* cells analyzed by flow cytometry (the fluorescence intensity measured for R800 WT cells labeled with the FDA issued from compound DIBO 8 pre-clicked to azido-AF647 was taken as the reference). (C and D) Conventional fluorescence microscopy images of R800 WT (C) or Δ*dacA* (D) cells labeled with FDA as illustrated in panel (A). Left panels show the peptidoglycan (PG) synthesis region revealed by the AF647 signal, middle panels show phase contrast (PC) images and right panels show merged phase contrast and AF647 channels. All images were recorded with the same acquisition parameters, and signal levels were treated identically for each panel. Scale bars, 1 μm.

To analyze the trimming action of PBP3 during pulse labeling, we used a R800 strain deleted from the *dacA* gene coding for PBP3 (Fig. 3A). When analyzed by flow cytometry after a 10 min pulse-labeling with FDAs, all Δ*dacA* cells exhibited stronger fluorescence compared to WT cells (Fig. 3B). Observation of the labeled cells by conventional fluorescence microscopy confirmed this result (Fig. 3C and D), highlighting a significant trimming action of PBP3, which varies depending on the incorporated FDA. The FDA made from d-Ala-DIBO 8 and azido-AF647 is less prone to be removed by PBP3 (2.9-fold reduction of the signal intensity between Δ*dacA* and WT cells, Fig. 3B) compared to d-Ala-BCN 7 and d-Ala-DBCO 8 products (8.8 and 5.1-fold reduction, respectively). The incorporated FDA obtained from d-Ala-DIBO 8 is thus the poorest substrate for PBP3. In the absence of PBP3, the strongest fluorescent signal was observed for the FDA obtained from d-Ala-BCN 7, indicating that TPs incorporate this compound more efficiently than those obtained from d-Ala-DIBO 8 or d-Ala-DBCO 9. Eventually, the best balance between FDA incorporation and trimming is obtained with the compound made from d-Ala-DIBO 8, as it provides the strongest fluorescent signal in WT cells (Fig. 2C and 3A).

**Fig. 4:**
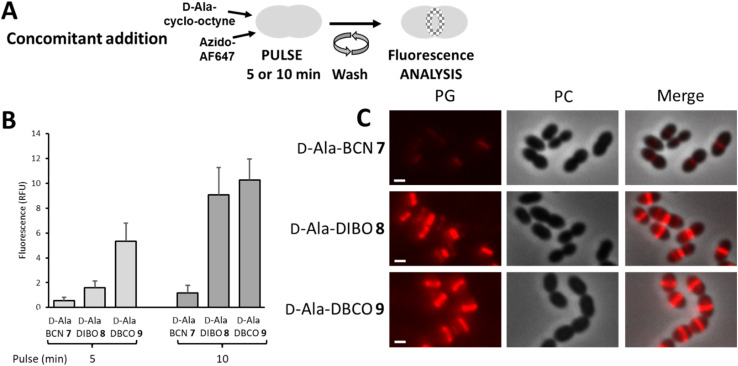
(A) “One-pot” two-step pulse-labeling scheme. *S. pneumoniae* D39 Δ*cps* cells were labeled by concomitant addition of d-Ala-cyclo-octyne (7–9) and azido-AF647. (B) Histogram showing the relative fluorescence intensity of D39 Δ*cps* cells labeled for 5 or 10 min and analyzed by flow cytometry. (C) Conventional fluorescence microscopy images of D39 Δ*cps* cells labeled for 10 min with products (7–9) and azido-AF647, as illustrated in panel (A). Left panels show the peptidoglycan (PG) synthesis region revealed by the AF647 signal, middle panels show phase contrast (PC) images and right panels show merged phase contrast and AF647 channels. All images were recorded with the same acquisition parameters, and signal levels were treated identically for each panel. Scale bars, 1 μm.

The “one-pot” two-step approach was next investigated with the concomitant incubation of d-Ala-cyclo-octyne 7–9 and azido-AF647 with D39 Δ*cps* cells, in the context of a 5- or 10 min pulse-labeling experiment (Fig. 4A). Since d-Ala-cyclo-octynes 7–9 carry fast SPAAC groups, their incorporation into the PG competes with their click reaction in solution with azido-AF647. The labeling thus results from the combination of a true two-step process (*i.e.* incorporation of d-Ala-cyclo-octyne into the PG, followed by click reaction with azido-AF647) and incorporation of FDA that has been formed in the medium by click reaction between free d-Ala-cyclo-octyne and azido-dye. Unlike the previous one-step labeling experiment using pre-clicked FDAs, the clickable probe that provided the strongest fluorescent signal was this time d-Ala-DBCO 9 (Fig. 4B). The difference observed between the two experiments suggests that during concomitant addition of d-Ala-cyclo-octyne and azido-AF647, the labeling mainly results from a two-step process, with rapid incorporation of the clickable d-Ala probes, followed by rapid fixation of the fluorescent dye. Between 5 and 10 min of pulse, the brightness of the labeling doubles for d-Ala-BCN 7 and d-Ala-DBCO 9, whereas it quadruples for d-Ala-DIBO 8, reaching almost the labeling level of d-Ala-DBCO 9. It is interesting to note that modified d-Ala that are poorly incorporated as pre-clicked FDA (one-step labeling), such as that produced from d-Ala-DBCO 9 (see Fig. 2D), can result in much stronger labeling when incorporated with a “one-pot” two-step approach.

**Fig. 5:**
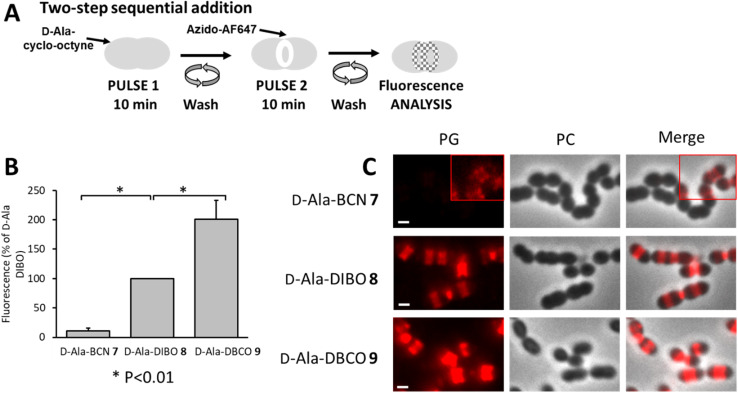
(A) Two-step pulse-labeling scheme. *S. pneumoniae* D39 Δ*cps* cells were incubated for 10 min with d-Ala cyclo-octyne (7–9), washed and labeled with azido-AF647 for 10 min. (B) Histogram showing the relative fluorescence intensity of labeled D39 Δ*cps* cells analyzed by flow cytometry. Fluorescence intensity obtained with d-Ala-DIBO 8 was taken as the reference. (C) Conventional fluorescence microscopy images of D39 Δ*cps* cells labeled with products 7–9 and azido-AF647 as illustrated in panel (A). Left panels show the peptidoglycan (PG) synthesis region revealed by the AF647 signal, middle panels show phase contrast (PC) images and right panels show merged phase contrast and AF647 channels. All images were recorded with the same acquisition parameters, and signal levels were treated identically for each panel. The red insets show regions of the images in which the contrast was modified to visualize the fluorescent signal. Scale bars, 1 μm.

To better separate the respective effects of the d-Ala incorporation and the click fluorescent labeling, the two-step approach was also performed sequentially, by incubating cells with products 7–9 for 10 min, followed by washes and labeling by click reaction with azido-AF647 for 10 min (Fig. 5A). In these conditions, the trimming took place over a longer time (20 min in total), while the incorporation of clickable d-Ala and the click reaction remained unchanged (10 min each). This longer trimming time had no significant influence on the ranking of labeling efficiency between the clickable d-Ala (DBCO > DIBO > BCN, Fig. 5B). With the sequential approach, the labeling efficiency observed for compounds 7–9 correlates with their SPAAC reaction kinetics.

**Fig. 6:**
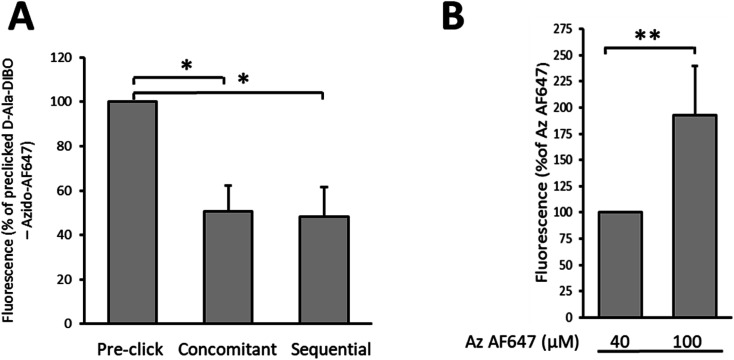
(A) Comparison of the fluorescence intensity of labeled *S. pneumoniae* D39 Δ*cps* cells analyzed by flow cytometry (*p < 0.05). Cells were labeled for 10 min with d-Ala-DIBO 8 and azido-AF647 by the one-step (pre-click), “one-pot” two-step (concomitant) or two-steps (sequential) approaches. (B) Comparison of the fluorescence intensity of labeled *S. pneumoniae* D39 Δ*cps* cells analyzed by flow cytometry. Cells were labeled for 10 min with d-Ala-DIBO 8 and 40 or 100 μM of azido-AF647 by the “one-pot” two-step (concomitant) approach. An increasing concentration of azido-AF647 improves the labeling (**p < 0.01).

Finally, we compared by flow cytometry the one-step, the “one-pot” two-step and the two-step labeling approaches to identify which one provides the most intense labeling for a 10 min pulse with d-Ala-DIBO 8 (Fig. 6A). The one-step approach using pre-clicked FDA appears to be the most effective, with twice the brightness compared to the other two approaches. The SPAAC reaction in the context of the “one-pot” two-step approach is therefore not fast enough during a 10 min pulse to match the fluorescence intensity obtained by one-step labeling with FDA. However, by increasing the concentration of the fluorescent dye to speed up the click reaction, it was possible to significantly increase the labeling rate and reach the efficiency of the one-step labeling with FDA (see comparison for DIBO, Fig. 6A and B).

### Comparison of labeling approaches in pulse-chase experiments

**Fig. 7:**
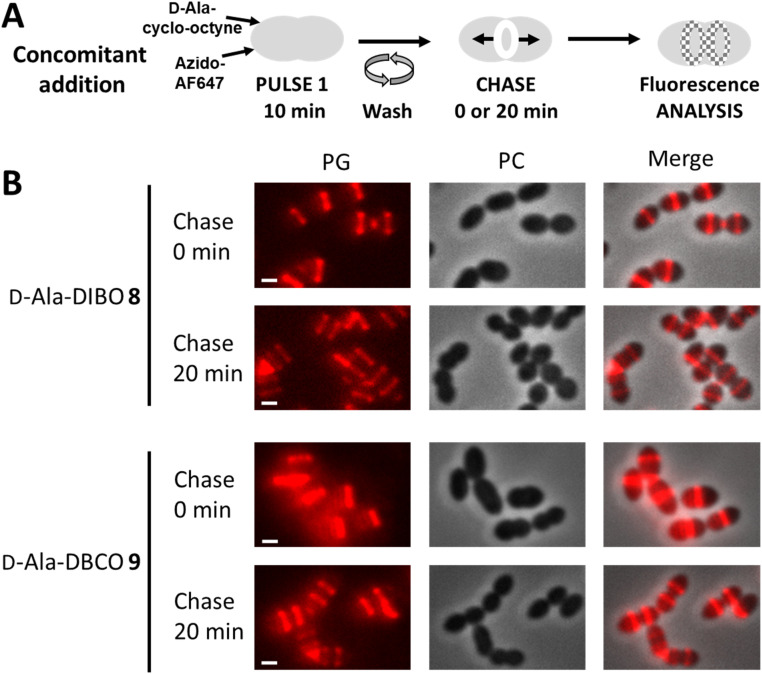
(A) “One-pot” two-step pulse-chase labeling scheme. (B) Conventional fluorescence microscopy images of *S. pneumoniae* D39 Δ*cps* cells labeled with products 8, 9 and azido-AF647 as illustrated in panel A, observed before or after a 20 min chase. Left panels show the peptidoglycan (PG) synthesis region revealed by the AF647 signal, middle panels show phase contrast images and right panels show merged phase contrast and AF647 channels. All images were recorded with the same acquisition parameters, and signal levels were treated identically for each panel. Scale bars, 1 μm.

We next investigated the labeling obtained with our d-Ala probes in a pulse-chase experiment, which allows investigating the fate of neosynthesized PG regions (Fig. 7A). Given that d-Ala-DIBO 8 and d-Ala-DBCO 9 provided the strongest signals in the “one-pot” two-step labeling, we used these probes and this approach to label D39 Δ*cps* cells for 10 min. The cells were then directly observed by conventional fluorescence microscopy, or further incubated for 20 min in the absence of the labeling compounds (chase period) before microscopy analysis (Fig. 7B). The labeling remained persistent after the chase period, indicating that the incorporated probes have not been significantly trimmed over time and can thus be used to investigate the localization of newly synthesized regions of the PG along the cell cycle.

### Two-color labeling of PG and TA for conventional optical microscopy

**Fig. 8:**
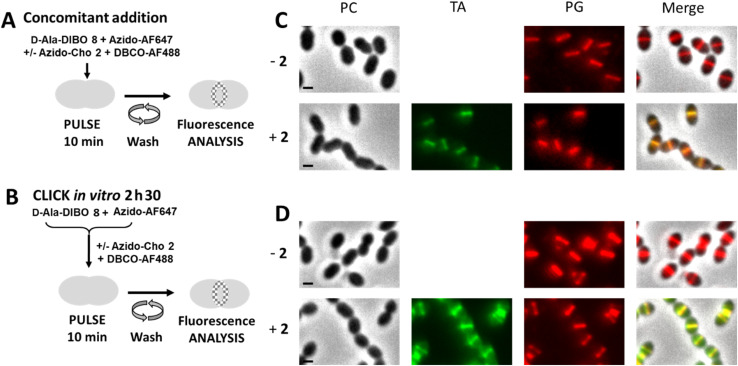
(A) “One-pot” two-step dual labeling scheme for PG and TA. *S. pneumoniae* R800 WT cells were incubated with d-Ala-DIBO ^8^, azido-AF647 and DBCO-AF488, in the presence or absence of azido-Cho 2 for 10 min. (B) Labeling scheme combining the one-step approach with FDA and “one-pot” two-step approach for TA. *S. pneumoniae* R800 WT cells were incubated with the FDA issued from d-Ala-DIBO 8 clicked to azido-AF647, and with DBCO-AF488, in the presence or absence of azido-Cho 2 for 10 min. (C and D). Conventional fluorescence microscopy images of R800 WT cells labeled as illustrated in panels (A) (for C) or (B) (for D), in the presence (+2) or absence (−2) of azido-Cho. Images acquired with the phase contrast (PC), AF488 (for labeled teichoic acids, TA) and AF647 (for labeled peptidoglycan, PG) channels are shown, together with a merged image. All images were recorded with the same acquisition parameters, and signal levels were treated identically for each panel scale bars, 1 μm.

The next objective was to carry out concomitant labeling of PG and TA, each with a different fluorescent probe. We have already performed such co-labeling during a 5 min pulse.^8^ It consisted of combining a one-step labeling of PG using the FDA hydroxycoumarin-amide-d-Ala (HADA) with a “one-pot” two-step labeling of TA using azido-Cho 2 and DIBO-AF488. However, as we have just seen, some FDAs do not efficiently label the PG when used in one-step labeling pulse (see Fig. 2), thus precluding the use of large fluorophores, which could more easily extend the color range and be compatible with super-resolved imaging by dSTORM. We thus tested a dual “one-pot” two-step approach, involving two SPAAC reactions at the same time (Fig. 8A), which is an easy procedure to implement rather than successive addition and washing steps. We know that choline can only be incorporated into TA if not clicked to a fluorophore^6^ and that the “one-pot” two-step labeling with clickable d-Ala involves predominantly the two-step pathway (see Fig. 4). In a dual “one-pot” two-step approach, the clickable choline and d-Alas should therefore be mostly incorporated into the cell wall before being conjugated to the fluorescent dyes or to each other. The main risk in such experiment lies in higher occurrence of unwanted SPAAC cross-reactions, *i.e.* (1) in solution, between clickable d-Ala and choline, which may adversely affect their availability to the cell, or between the latter and the clickable dye in the second step, and (2) in the cell wall, where incorporated clickable d-Ala and choline may react with probes remaining present in solution or with probes incorporated in neighboring TA or PG, leading to impaired cell wall synthesis and growth arrest. To test the innocuity of complementary clickable functions in TA and PG, D39 Δ*cps* cells were incubated with a mixture of azido-Cho 2 and/or d-Ala-DIBO 8 for 10 min, washed and further grown in BHI to assess the ability of labeled cells to resume growth. Incorporation of azido-Cho 2 and d-Ala-DIBO 8 for 10 min had no effect on the bacterial growth (Fig. S2†). This observation suggests that complementary clickable d-Ala and choline probes do not create any toxic artificial cross-link in the cell wall. We cannot exclude that some labeled PG and TA may click together but if this were to happen, it does not prevent cell growth, as previously reported for artificial cross-linking of the PG.^16–18^

Cells labeled with the “one-pot” two-step approach were then analyzed by conventional fluorescence microscopy (Fig. 8C). In parallel, we combined one-step labeling of PG using FDA (resulting from a pre-click reaction between d-Ala-DIBO 8 and azido-AF647) with “one-pot” two-step labeling of TA (Fig. 8B). Both experiments showed consistent labeling of PG and TA, but higher levels of fluorescence were obtained with the “one-pot” two-step approach (Fig. 8D). For the “one-pot” two-step approach, an experiment carried out without azido-Cho 2 (−2) showed an intensity of PG labeling that was similar to that obtained with all the labeling compounds, indicating that the expected cross-reaction between azido-AF647 and DBCO-AF488 does not impair fluorescent labeling. Although DIBO has slower reaction kinetics than DBCO, the click reaction between d-Ala-DIBO 8 and azido-AF647 is fast enough to not be significantly impacted by the cross-reaction between azido-AF647 and DBCO-AF488. Altogether, these results show that a dual “one-pot” two-step approach with complementary PG and TA probes allows efficient two-color labeling.

### Two-color labeling of PG and TA for dSTORM microscopy

**Fig. 9:**
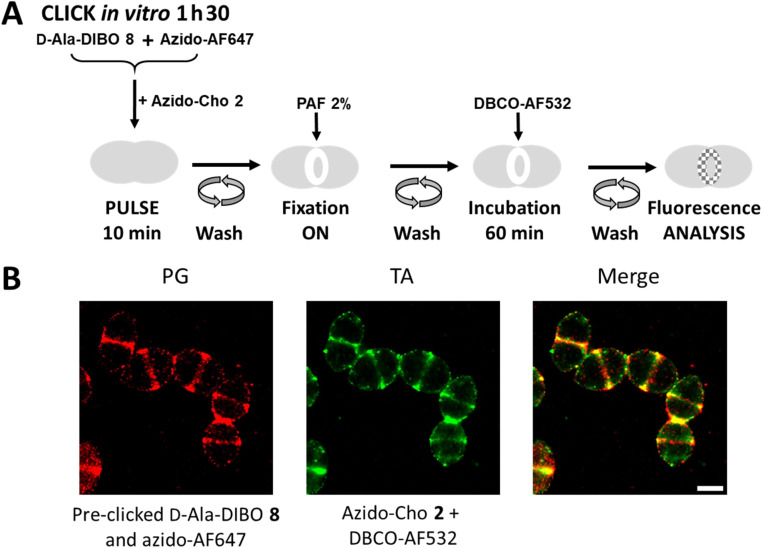
(A) Hybrid labeling scheme for dual localization of PG and TA. Cells were incubated for 10 min in the presence of FDA (d-Ala-DIBO 8 pre-clicked with azido-AF647) and azido-choline 2, chemically fixed with paraformaldehyde and incubated with DBCO-AF532 for 60 min. (B) Super-resolution dSTORM images of *S. pneumoniae* D39 Δ*cps* cells in which PG and TA were co-labeled as illustrated in panel A. Reconstructed dSTORM images obtained from the AF647 (PG labeling) and AF532 (TA labeling) channels are shown, together with a merged image. Scale bar, 1 μm.

To perform dSTORM, cells must be fixed to prevent cell lysis, which is induced by the phototoxicity of the lasers used for sample illumination. Cell fixation offers the possibility to dissociate certain labeling steps. For PG labeling, FDA derived from the assembly of d-Ala-DIBO 8 with azido-AF647 was used, as being previously identified as the most effective (Fig. 2). For TA labeling, the two-step approach is mandatory. FDA and azido-Cho 2 were thus incubated at the same time with the bacteria for 10 min (Fig. 9A). After washing, the cells were fixed overnight before incubation with DBCO-AF532 for fluorescent labeling of the incorporated azido-Cho 2. As the cells were fixed, the click reaction between DBCO-AF532 and azido-Cho 2 could be carried out for a longer time (60 min) than in a “one-pot” two-step experiment, without altering the temporal resolution of the labeling. This strategy provided equivalent labeling densities between PG and TA (Fig. 9B). In these conditions, we have succeeded in obtaining the first two-color dSTORM images of PG and TA synthesis regions. The experiment consisting of interchanging the two fluorescent probes, *i.e.* using FDA derived from the assembly of d-Ala-DIBO 8 with azido-AF532 and labeling TA with DBCO-AF647, was also carried out successfully (Fig. S3†).

## Conclusions

Fluorescent labeling of cell wall components requires identifying suitable probes and methods to obtain the best temporal and spatial resolution, or in other words to obtain an intense fluorescent signal for a minimal labeling time. In this work, we tested the labeling efficiency of three clickable d-Ala 7–9 in various experimental conditions. Our data show that when incorporation of FDA by TP proves difficult, it is possible to circumvent this issue with “one-pot” two-step labeling, *i.e.* where the clickable d-Ala is inserted into the PG before its conjugation to the fluorescent dye that is also present in the reaction mix. In our hands, this observation was particularly true for d-Ala-DBCO 9. We have also demonstrated the effectiveness of this approach for two-color “one-pot” two-step labeling of PG and TA, which involves two simultaneous SPAAC reactions. In this approach, the SPAAC reaction must be rapid to achieve strong fluorescent labeling in a short time, but rapid click kinetics can also lead to the formation of probe-fluorophore conjugates that will not be well incorporated into the cell wall. A balance must therefore be found between the kinetics of the click reaction and the incorporation of the probe. In our work, we show that d-Ala-DIBO 8 and d-Ala-DBCO 9, which harbor rapid SPAAC kinetics, are nonetheless incorporated into the PG before being clicked to the fluorophore, indicating that the labeling mainly occurs in a two-step process. This method is simple to implement, compatible with pulse-chase studies on living cells, and offers a relatively wide choice of fluorophores that can be conjugated by click reaction. The question is whether this “one-pot” two-step method can be applied to Gram-negative bacteria, given that they have an additional external lipid membrane, bringing more constraints to the incorporation of modified metabolites and clickable fluorescent probes. Success will depend on identifying the best operating conditions for matching the size and kinetic parameters of the clickable groups. Regarding the labeling conditions for dSTORM, the FDA obtained from the coupling of d-Ala-DIBO 8 with azido-AF647 was identified as the best compound for one-step labeling. Combined with a sequential two-step labeling of TA in which the SPAAC reaction is carried out over a long period on fixed cells, this approach enabled us to obtain the first super-resolved co-localization images of PG and TA. This protocol lays experimental foundations for the study of the interplay between the synthesis mechanisms of the two main components of the pneumococcal cell wall.

## Data availability

We declare that all the data presented in this work are available in the ESI† submitted with this manuscript. Raw data are available on the Zenodo platform.

## Author contributions

MB, AC, MN, AZ and CD performed experiments; AZ, BP, CM, CD and YSW designed research; MB, AC, MN, AZ, BP, CD and YSW analyzed data; MB, CM, CD and YSW wrote the manuscript; AZ, CM, CD and YSW raised funds.

## Conflicts of interest

The authors declare no competing interests.

## Supplementary Material

RA-014-D4RA04945D-s001
