# Supplementary material for: To click or not to click for short pulse-labeling of the bacterial cell wall
Source: RSC Adv. 2024 Oct 21;14(45):33133–42. doi: 10.1039/d4ra04945d (PMC11492190; doi:10.1039/d4ra04945d)

Supplementary Information (SI) for RSC Advances.
This journal is © The Royal Society of Chemistry 2024

To click or not to click for short pulse-labeling of the bacterial cell wall

Morgane Baudoin,[a] Anne Chouquet,[b] Mai Nguyen,[b] André Zapun,[b] Basile Pérès,[a] Cécile Morlot,[b] Claire Durmort,*[b] Yung-Sing Wong *[a]

[a] Univ. Grenoble Alpes, CNRS, DPM, 38000 Grenoble, France.

[b] Univ. Grenoble Alpes, CNRS, CEA, IBS, 38000 Grenoble, France

Table of contents

1. Supplementary schemes and figures........2

2. General synthetic methods 3

3. PG labelling in *S. pneumoniae* 13

4. SPAAC kinetics study 14

5. References........23

6. Appendix........25

1. Supplementary schemes and figures

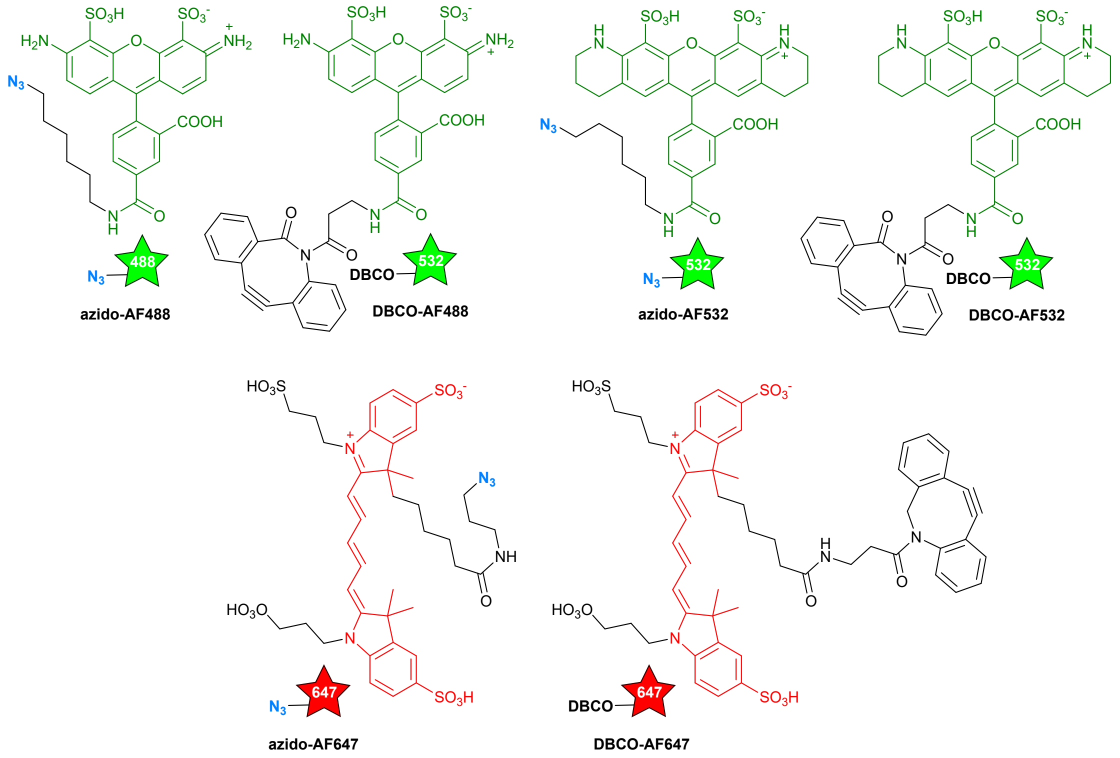

Fig. S1. Molecular structures of commercially available clickable fluorescent dyes used in this work.

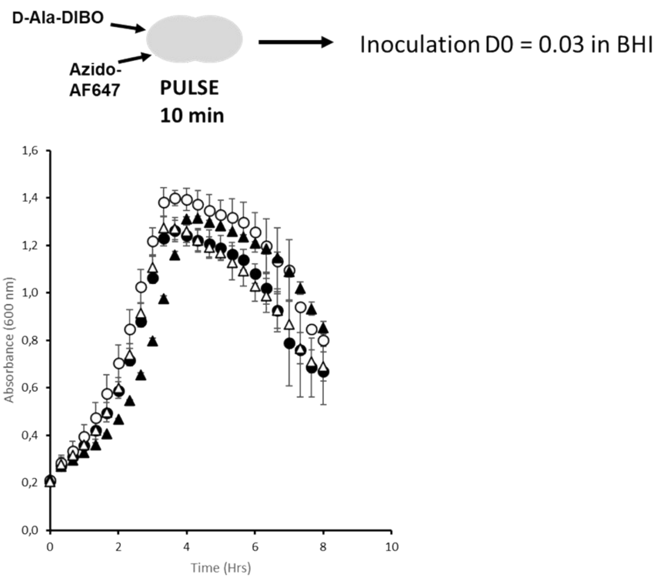

Fig. S2. Comparison of the growth of *S. pneumoniae* D39 Δ*cps* cells after a 10-min incubation with 250 µM of ● choline chloride, ○ azido-choline **2**, ▲ D-Ala-DIBO **8** or Δ azido-choline **2** and D-Ala DIBO **8**. Absorbance at 600 nm was monitored every 20 min.

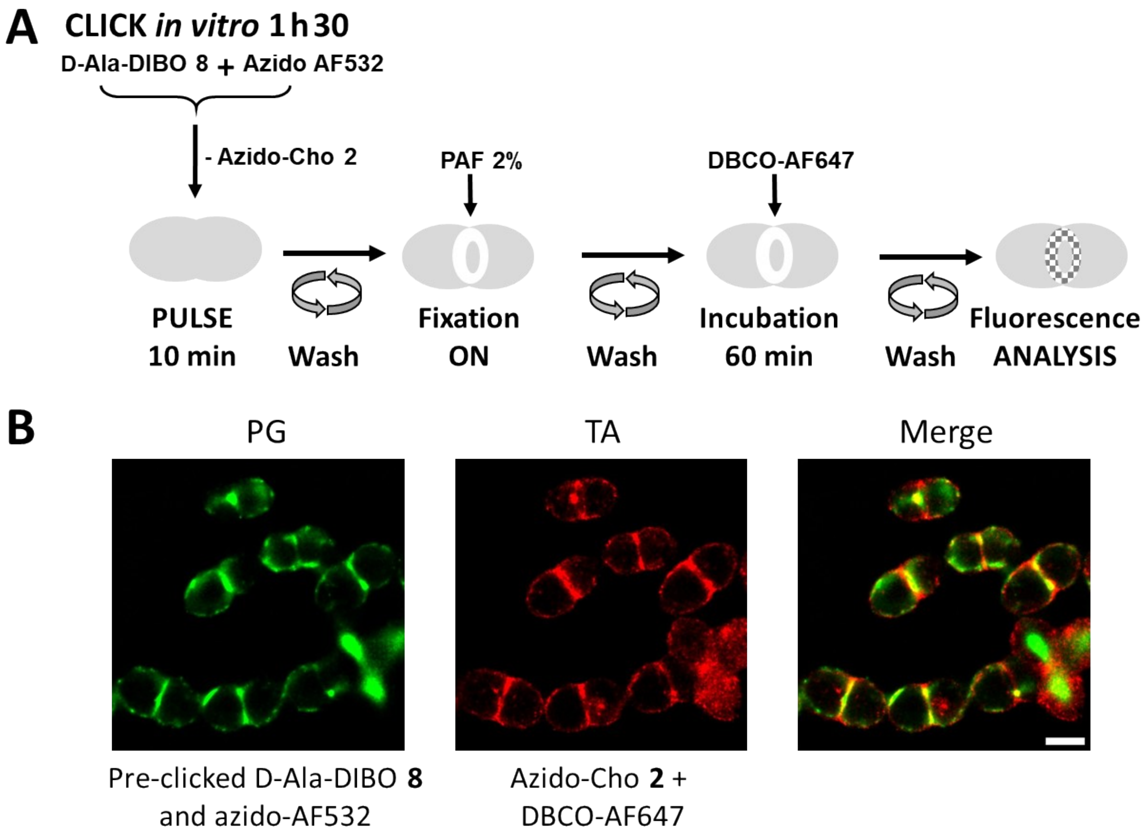

Fig. S3. Hybrid labeling scheme for dual localization of PG and TA. Cells were incubated for 10 min in the presence of FDA (D-Ala-DIBO **8** pre-clicked with azido-AF532) and azido-choline **2**, chemically fixed with paraformaldehyde and incubated with DBCO-AF647 for 60 min. **B.** Super-resolution dSTORM images of *S. pneumoniae* D39 Δ*cps* cells in which PG and TA were co-labeled as illustrated in panel A. Reconstructed dSTORM images obtained from the AF532 (PG labeling) and AF647 (TA labeling) channels are shown, together with a merged image. Scale bar, 1 µm.

2. General synthetic methods

Chemicals and solvents used for reactions were purchased from BLD-Pharm, Fluorochem, Merck and Sigma-Aldrich and were used without any other purification.

The reactions were monitored by thin-layer chromatography (TLC) on Macherey-Nagel silica gel 60 with UV detection at 254 nm. Flash column chromatographies (FCC) were performed using Macherey-Nagel silica gel 60 M.

HPLC analyses were performed on an Agilent 1100 Series equipped with a column Macherey-Nagel EC 250/ 4.6 Nucleodur C18 Isis (5 µm, 250 × 4.6 mm). The retention time (t_R) is expressed in minutes with a flow rate of 1 mL/min.

Nuclear magnetic resonance (NMR) spectra were recorded either on a Brüker Avance Neo (400 MHz for ^{1}H, 100 MHz for ^{13}C) or on a Brüker Avance III (500 MHz for ^{1}H, 126 MHz for ^{13}C). Deuterated solvents (MeOD, DMSO-d_6 and $(CD_3)_2CO$) were purchased from Deutero GmbH (Kastellaun, Germany). Chemical shifts (δ) are expressed in ppm (parts per million) with the following references: MeOD (3.30 ppm for ^{1}H and 49.15 ppm for ^{13}C), DMSO-d_6 (2.50 ppm for ^{1}H and 39.52 ppm for ^{13}C) and $(CD_3)_2CO$ (2.05 ppm for ^{1}H and 29.84 ppm for ^{13}C). The following abbreviations were used: s (singlet), br s (broad singlet), d (doublet), m (multiplet). When two signals are observed for protons on the same carbon atom close to a stereocenter, they are differenciated with the letters a and b. In presence of rotamers or diastereoisomers, when two signals are observed for the same proton or carbon, the second signal is labeled with a *.

High resolution mass spectrometry (HRMS) spectra were recorded by the Institut de Chimie Moléculaire de Grenoble (ICMG). The spectra were recorded on a LTQ Orbitrap XL Thermo Scientific with ElectroSpray Ionization (ESI).

Optical rotations were recorded on a Perkin Elmer Polarimeter 341 (wavelength 589 nm) on a sodium lamp using a cuvette with a path length of 10 mm. Specific optical rotations are expressed in deg·mL·g^{-1}·dm^{-1} and reported without unit. The associated concentration is given in g/(100 mL).

11,12-Didehydro-5,6-dihydrodibenzo[*a*,*e*][8]annulen-5-yl 4-nitrophenyl carbonate was prepared according to the literature.[1] Succinimidyl 4-(11,12-didehydrodibenzo[*b*,*f*]azocin-5(6*H*)-yl)-4-oxobutanoate (DBCO-NHS) was prepared according to the literature.[3] BCN-D-Ala was synthesized as described by Bertozzi *et al.*[2]

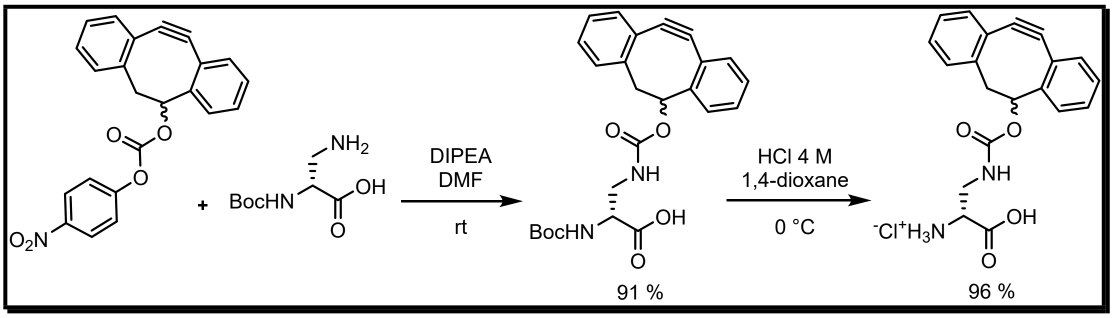

<u>(*R*)-2-((*tert*-Butoxycarbonyl)amino)-3-((11,12-didehydro-5,6-dihydrodibenzo[*a*,*e*][8]annulen-5-yloxycarbonyl)amino)propanoic acid</u>

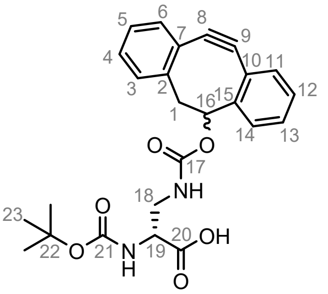

Chemical Formula: $C_{25}H_{26}N_2O_6$
Molecular Weight: 450,49

N-Boc-D-Ala-DIBO

To a solution of DIBO-(4-nitrophenyl) carbonate (84 mg, 0.22 mmol, 1 equiv.) in DMF (5 mL) was added DIPEA (380 µL, 2.2 mmol, 10 equiv.) and Boc-D-2,3-diaminopropionic acid (49 mg, 0.24 mmol, 1.1 equiv.) at room temperature. After 15 h, DMF was removed under vacuum and an aqueous solution of H_3PO_4 (5 % H_3PO_4 in water) was added. The resulting mixture was extracted with EtOAc and the combined organic layers were dried over $MgSO_4$, filtered and concentrated under reduced pressure. The crude product was purified by FCC (0.3 % AcOH/ 2.7 % MeOH/ 97 % CH_2Cl_2 to 0.5 % AcOH/ 4.5 % MeOH/ 95 % CH_2Cl_2) to afford the protected amino acid ***N*-Boc-D-Ala-DIBO** as a white solid (90 mg, 91 % yield).

R_f = 0.21 (MeOH 6 %/ CH_2Cl_2 94 %)

^{1}H NMR (400 MHz, **$(CD_3)_2CO$**) δ (ppm) 1.42 (br s, 9H, H23), 2.84 (m, 1H, H1a), 3.24 (m, 1H, H1b), 3.54 (m, 1H, H18a), 3.65 (m, 1H, H18b), 4.39 (m, 1H, H19), 5.46 (m, 1H, H16), 7.32-7.52 (m, 7H, H_{Arom}), 7.63 (m, 1H, H_{Arom}); **^{13}C NMR** (100 MHz, **$(CD_3)_2CO$**) δ (ppm) 28.6 (3×CH_3, 3×C23), 43.1 (CH_2, C18), 47.0 (CH_2, C1), 55.1 (CH, C19), 77.3 (CH, C16), 79.5 (C, C22), 110.7 (C, C9), 113.4 (C, C8), 121.8 (C, C10), 124.4 (C, C7), 125.1 (CH, C_{Arom}), 126.6 (CH, C_{Arom}), 126.9 (CH, C_{Arom}), 128.0 (CH, C_{Arom}), 128.1 (CH, C_{Arom}), 129.1 (CH, C_{Arom}), 131.2 (CH, C_{Arom}), 131.3 (CH, C_{Arom}), 152.2 (C, C2), 153.3 (C, C15), 156.5 (C, C17 or C21), 156.9 (C, C17 or C21), 172.4 (C, C20)

HRMS (ESI): *m/z* calc. for $C_{25}H_{26}N_2O_6$ $[M-H]^+$ 449.1718, found 449.1713

$[\alpha]_D^{20}$ = + 6.86 (c = 0.91, MeOH)

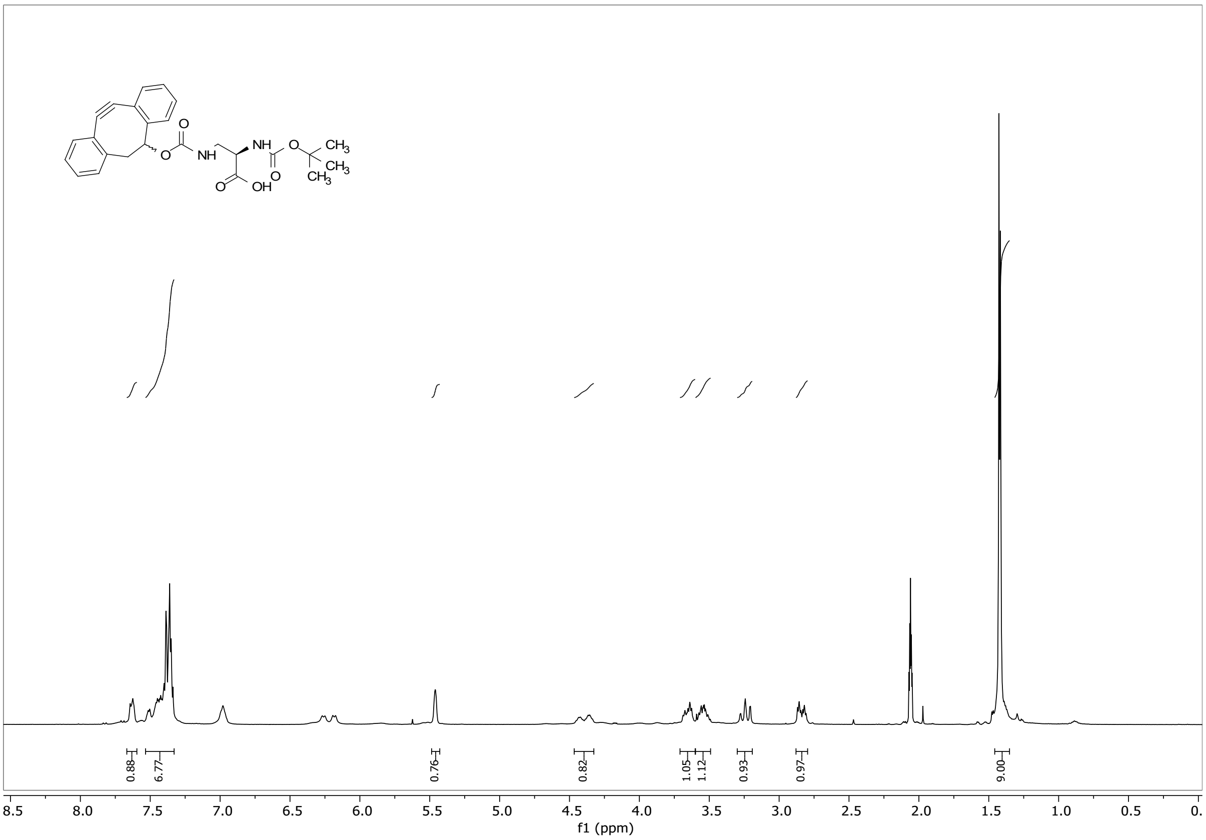

0.88
6.77
0.76
0.82
1.05
1.12
0.93
0.97
9.00
8.5 8.0 7.5 7.0 6.5 6.0 5.5 5.0 4.5 4.0 3.5 3.0 2.5 2.0 1.5 1.0 0.5 0.
f1 (ppm)

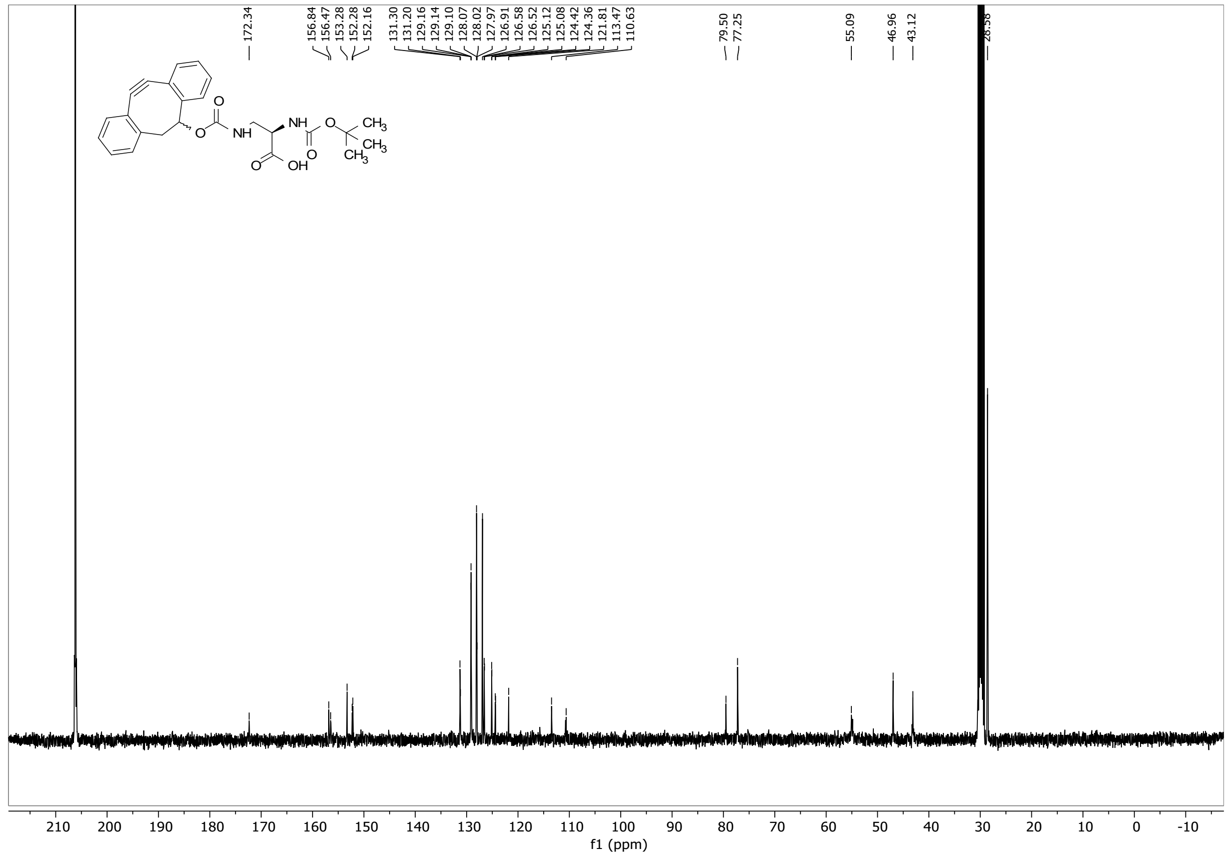

172.34
156.84
156.47
153.28
152.28
152.16
131.30
131.20
129.16
129.14
129.10
128.07
128.02
127.97
126.91
126.58
126.52
125.12
125.08
124.42
124.36
121.81
113.47
110.63
79.50
77.25
55.09
46.96
43.12
28.58
210 200 190 180 170 160 150 140 130 120 110 100 90 80 70 60 50 40 30 20 10 0 -10
f1 (ppm)

(*R*)-2-Amino-3-((11,12-didehydro-5,6-dihydrodibenzo[*a*,*e*][8]annulen-5-yloxycarbonyl)amino) propanoic acid

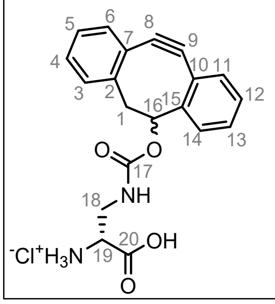

Chemical Formula: $C_{20}H_{19}ClN_2O_4$
Molecular Weight: 386,83

8

***N*-Boc-D-Ala-DIBO** (30 mg, 0.07 mmol, 1 equiv.) was dissolved in a solution of HCl (0.5 mL of a 4N solution in dioxane) and left under stirring during 1 h at 0 °C. The solvent was evaporated under vacuum and allowed to dry under high vacuum (oil pump). The resulting mixture was triturated in diethyl ether overnight. Then, the product was filtered and dried under vacuum to obtain the amino acid **8** as a white solid (24 mg, 96 %). After trituration in Et_2O, an aliquot was purified by reverse-phase chromatography on silica gel C18 (99,9 % H_2O/ 0.1 % TFA to 79.9 % H_2O/ 20 % MeOH/ 0.1 % TFA)

HPLC: t_R= 13.7 min (MeCN 30 %/ H_2O 70 %)

^{1}H NMR (400 MHz, **CD_3OD**) δ (ppm) 2.83 (m, 1H, H1a), 3.23 (m, 1H, H1b), 3.63 (m, 1H, H18a), 3.74 (m, 1H, H18b), 4.09 (m, 1H, H19), 5.45 (m, 1H, H16), 7.27-7.43 (m, 7H, H_{Arom}), 7.53-7.63 (m, 1H, H_{Arom}); **^{13}C NMR** (100 MHz, **CD_3OD**) δ (ppm) 42.1 (CH_2, C18), 47.1 (CH_2, C1) , 47.4 (CH_2, C1*), 55.1 (CH, C19), 78.9 (CH, C16), 110.1 (C, C9), 114.0 (C, C8), 122.5 (C, C10), 125.1 (C, C7), 125.2 (CH, C_{Arom}), 125.3 (CH, C_{Arom}), 127.0 (CH, C_{Arom}), 127.3 (CH, C_{Arom}), 128.4 (CH, C_{Arom}), 128.5 (CH, C_{Arom}), 129.4 (CH, C_{Arom}), 131.4 (CH, C_{Arom}), 152.5 (C, C2), 153.2 (C, C15), 158.9 (C, C17), 170.1 (C, C20)

HRMS (ESI): *m/z* calc. for $C_{20}H_{19}N_2O_4$ $[M]^+$ 351.1339, found 351.1339

$[\alpha]_D^{20}$ = + 11.71 (c = 0.85, MeOH)

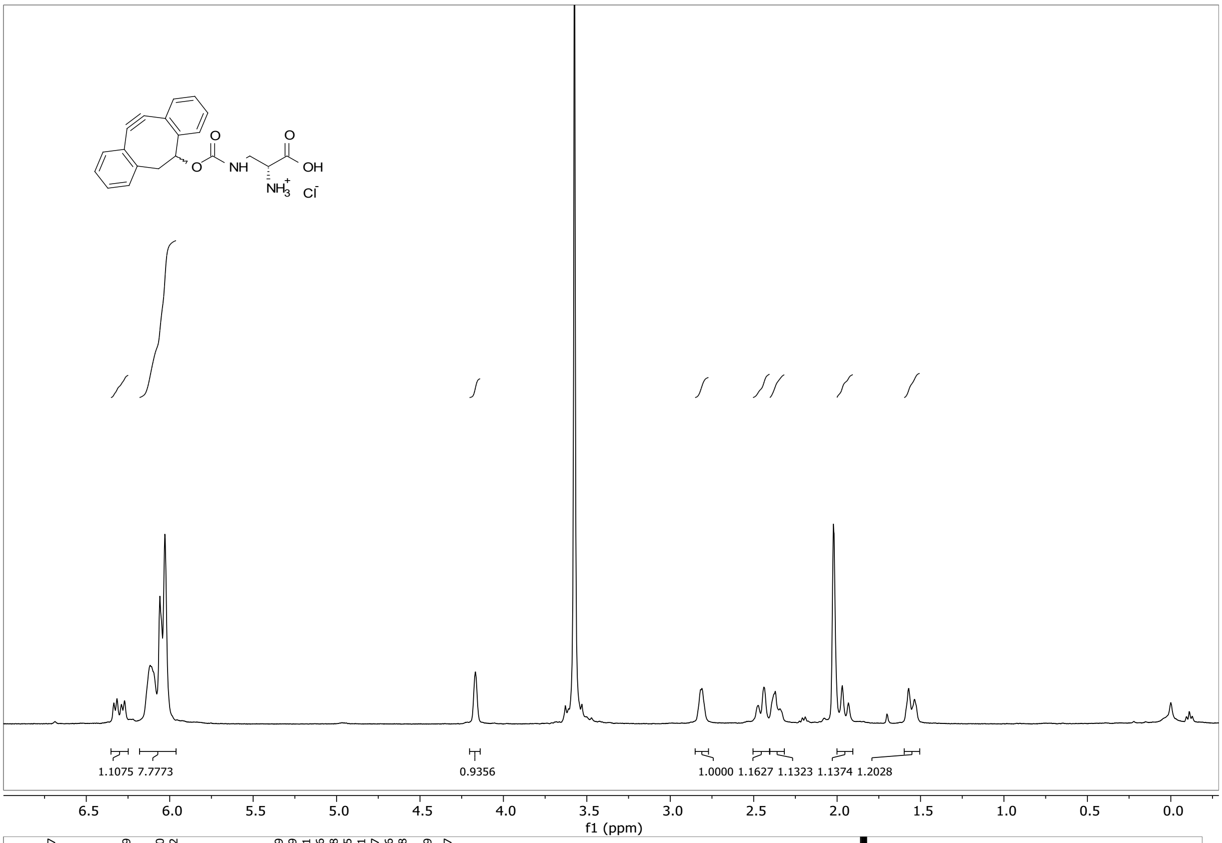
O
NH
OH
NH3+
Cl-
1.1075 7.7773
0.9356
1.0000 1.1627 1.1323 1.1374 1.2028
6.5 6.0 5.5 5.0 4.5 4.0 3.5 3.0 2.5 2.0 1.5 1.0 0.5 0.0
f1 (ppm)

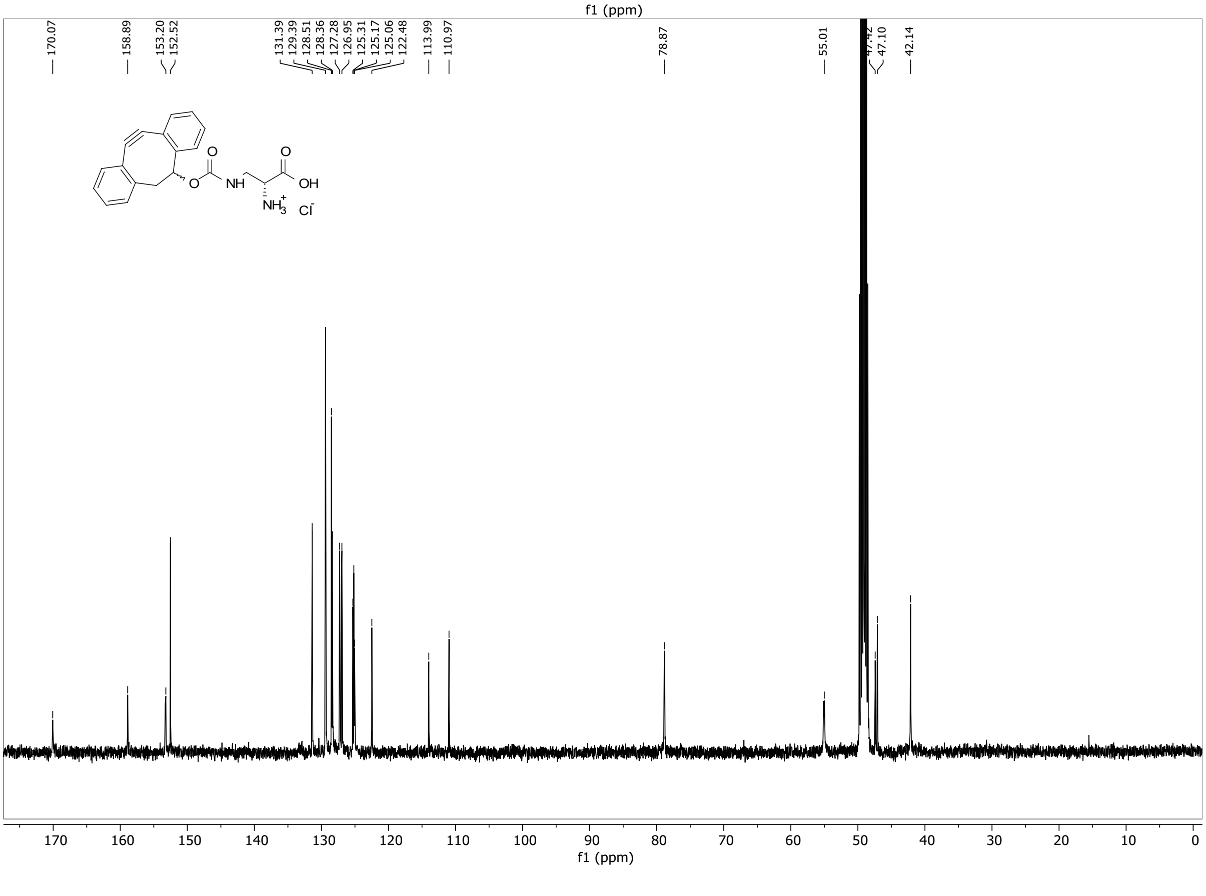
170.07
158.89
153.20
152.52
131.39
129.39
128.51
128.36
127.28
126.95
125.31
125.17
125.06
122.48
113.99
110.97
78.87
55.01
47.10
42.14
O
NH
OH
NH3+
Cl-
170 160 150 140 130 120 110 100 90 80 70 60 50 40 30 20 10 0
f1 (ppm)

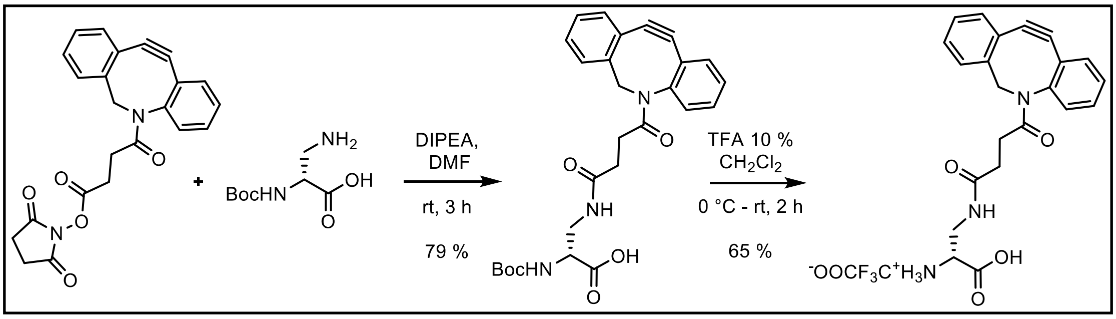

(*R*)-2-((*tert*-Butoxycarbonyl)amino)-3-(4-(11,12-didehydrodibenzo[*b*,*f*]azocin-5(6*H*)-yl)-4-oxobutanamido)propanoic acid

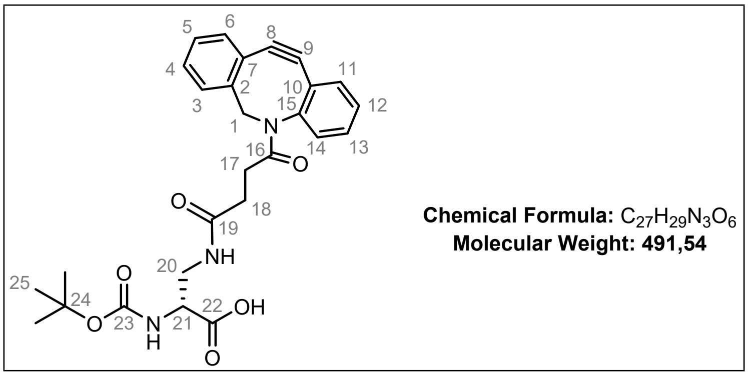

***N*-Boc-D-Ala-DBCO**

To a solution of activated DBCO-NHS (60 mg, 0.15 mmol, 1 equiv.) in DMF (4 mL) was added Boc-D-2,3-diaminopropionic acid (34 mg, 0.16 mmol, 1.1 equiv.) and DIPEA (114 µL, 0.66 mmol, 4.4 equiv.) at room temperature. After 3 h, DMF was removed under vacuum and an aqueous solution of H_3PO_4 (5 % H_3PO_4 in water) was added. The resulting mixture was extracted with EtOAc and the combined organic layers were dried over $MgSO_4$, filtered and concentrated under reduced pressure. The crude product was purified by FCC (0.3 % AcOH/ 2.7 % MeOH/ 97 % CH_2Cl_2) to afford the protected amino acid ***N*-Boc-D-Ala-DBCO** as a white solid (58 mg, 79 %).

R_f = 0.23 (AcOH 0.5 %/ MeOH 4.5 %/ CH_2Cl_2 95 %)

^{1}H NMR (400 MHz, **$(CD_3)_2CO$,** mixture of 2 rotamers) δ (ppm) 1.39 (s, 18H, 9×H25-9×H25*), 1.87 (m, 2H, H17), 2.09 (m, 2H, H18), 2.39 (m, 2H, H18*), 2.76 (m, 2H, H17*), 3.40-3.60 (m, 4H, H20a-H20a*-H20b-H20b*), 3.63 (d, J = 13.9 Hz, 2H, H1), 4.17-4.26 (m, 2H, H21-H21*), 5.14 (d, J = 13.9 Hz, 2H, H1*), 7.22-7.52 (m, 14H, 7×H_{Arom}-7×H_{Arom}*), 7.64-7.71 (m, 4H, H_{Arom}-H_{Arom}*-NH-NH*); **^{13}C NMR** (100 MHz, **$(CD_3)_2CO$,** mixture of rotamers) δ (ppm) 28.6 (6×CH_3, 3×C25-3×C25*), 30.8 (2×CH_2, C17-C17*), 31.46 (CH_2, C18), 31.51 (CH_2, C18*), 41.3 (2×CH_2, C20-C20*), 55.1 (2×CH, C21-C21*), 56.0 (2×CH_2, C1-C1*), 79.4 (2×C, C24-C24*), 108.9 (2×C, C9-C9*), 115.1 (2×C, C8-C8*), 123.0 (2×C, C10-C10*), 124.1

(2×C, C7-C7*), 126.0 (2×CH, C_{Arom}-C_{Arom}*), 127.67 (CH, C_{Arom}), 127.69 (CH, C_{Arom}*), 128.4 (2×CH, C_{Arom}-C_{Arom}*), 128.74 (CH, C_{Arom}), 128.77 (CH, C_{Arom}*), 128.89 (CH, C_{Arom}), 128.91 (CH, C_{Arom}*), 129.7 (2×CH, C_{Arom}-C_{Arom}*), 130.55 (CH, C_{Arom}), 130.59 (CH, C_{Arom}*), 133.5 (2×CH, C_{Arom}-C_{Arom}*), 149.5 (2×C, C2-C2*), 152.82 (C, C15), 152.86 (C, C15*), 156.4 (2×C, C23-C23*), 172.26 (2×C, C16 or C19 or C22- C16* or C19* or C22*), 172.34 (2×C, C16 or C19 or C22- C16* or C19* or C22*), 173.6 (2×C, C16 or C19 or C22- C16* or C19* or C22*)

HRMS (ESI): *m/z* calc. for $C_{27}H_{30}O_6N_3$ $[M+H]^+$ 492.2129, found 492.2125

$[\alpha]_D^{20}$ = + 5.2 (c = 1, $CHCl_3$)

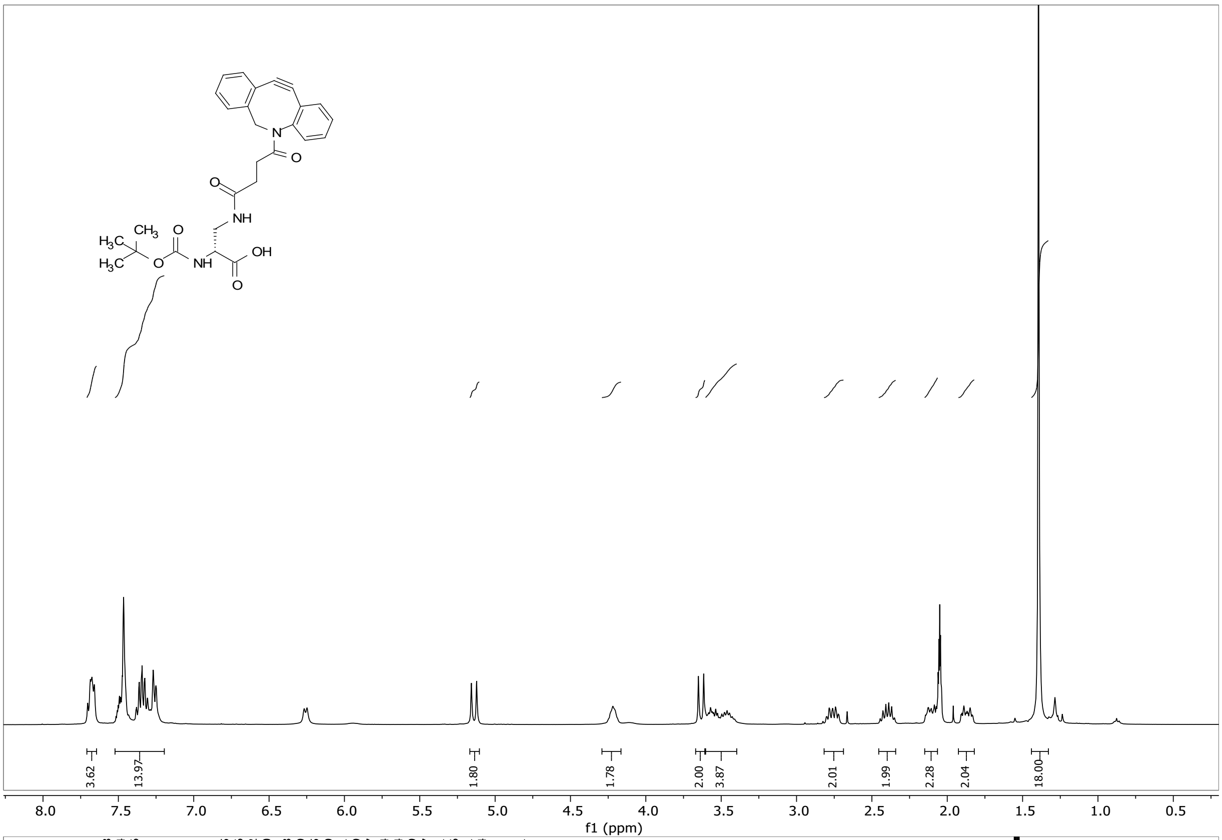

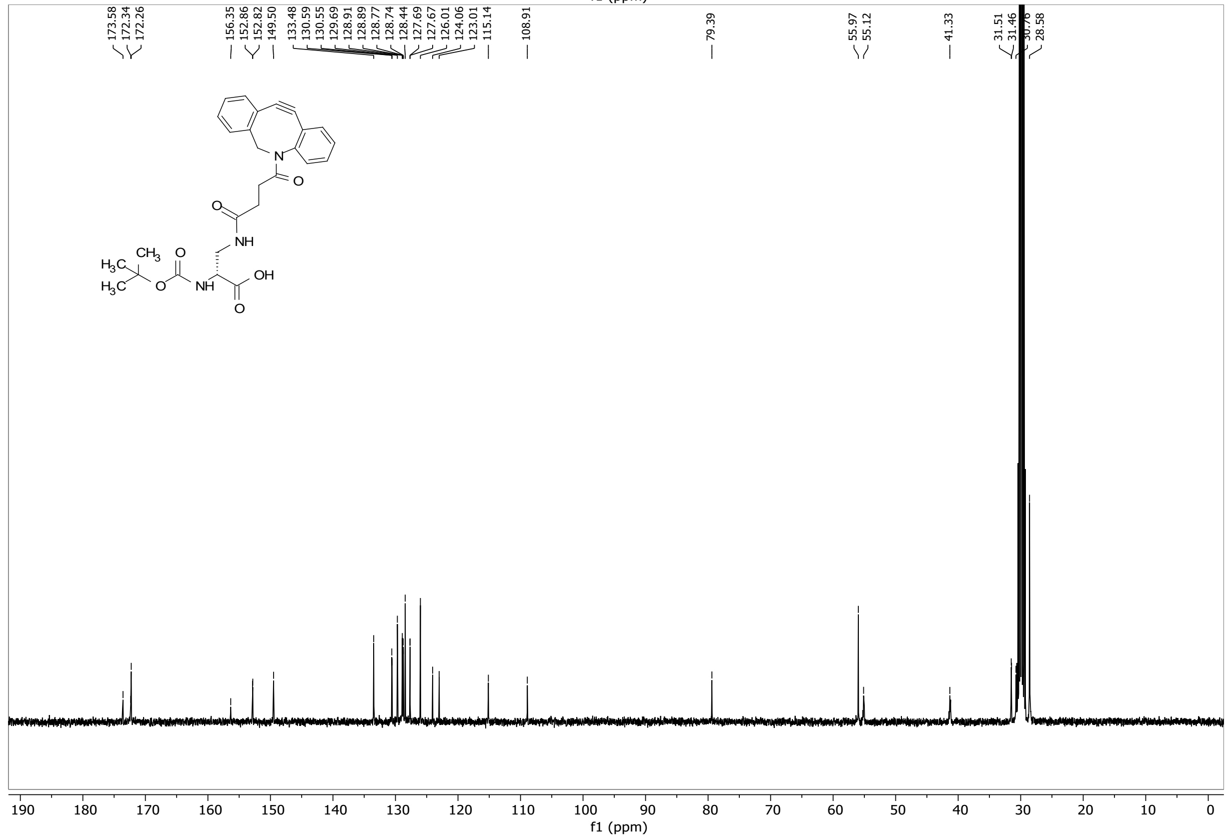

(*R*)-1-Carboxy-2-(4-(11,12-didehydrodibenzo[*b*,*f*]azocin-5(6*H*)-yl)-4-oxobutanamido)ethan-1-aminium 2,2,2-trifluoroacetate

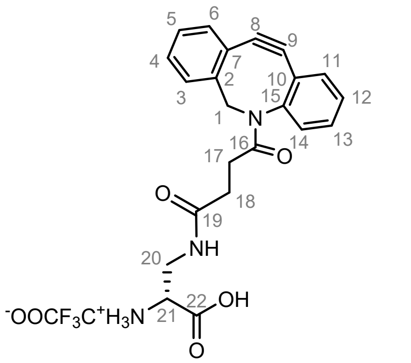

9

To a solution of protected ***N*-Boc-D-Ala-DBCO** (27.5 mg, 0.056 mmol) in 1.2 mL of CH_2Cl_2 was added TFA (300 µL) at 0 °C. After 1 h at 0 °C followed by 1 h at room temperature, CH_2Cl_2 and TFA were evaporated under vacuum and allowed to dry under high vacuum (oil pump). The resulting mixture was triturated in diethyl ether overnight and the product was filtered and dried under vacuum to obtain the amino acid **9** as an off-white solid (18.4 mg, 65 %). After trituration in Et_2O, an aliquot was purified by reverse-phase chromatography on silica gel C18 (99,9 % H_2O/ 0.1 % TFA to 79.9 % H_2O/ 20 % MeOH/ 0.1 % TFA)

HPLC: t_R= 5.7 min (MeCN 30 %/ H_2O 70 %)

^{1}H NMR (500 MHz, **DMSO-*d$_6$***, mixture of rotamers) δ (ppm) 1.82 (m, 2H, H17), 2.05 (m, 2H, H18), 2.26 (m, 2H, H18*), 2.61 (m, 2H, H17*), 3.18-3.28 (m, 2H, H20a-H20b), 3.39-3.52 (m, 4H, H21-H21*-H20a*-H20b*), 3.63 (d, *J* = 14.0 Hz, 2H, H1), 5.04 (d, *J* = 14.0 Hz, 2H, H1*), 7.29-7.41 (m, 6H, 3×H_{Arom}-3×H_{Arom}*), 7.45-7.53 (m, 6H, 3×H_{Arom}-3×H_{Arom}*), 7.62-7.70 (m, 4H, 2×H_{Arom}-2×H_{Arom}*); **^{13}C NMR** (126 MHz, **DMSO-*d$_6$***, mixture of rotamers) δ (ppm) 9.52 (CH_2, C17), 29.57 (CH_2, C17*), 30.33 (CH_2, C18), 30.37 (CH_2, C18*), 39.2 (2×CH_2, C20-C20*), 53.5 (2×CH, C21-C21*), 54.9 (2×CH_2, C1-C1*), 108.1 (2×C, C9-C9*), 114.2 (2×C, C8-C8*), 121.4 (2×C, C10-C10*), 122.5 (2×C, C7-C7*), 125.2 (2×C, C_{Arom}-C_{Arom}*), 126.8 (2×C, C_{Arom}-C_{Arom}*), 127.7 (C, 2×C_{Arom}-C_{Arom}*), 128.0 (2×C, C_{Arom}-C_{Arom}*), 128.2 (2×C, C_{Arom}-C_{Arom}*), 129.0 (2×C, C_{Arom}-C_{Arom}*), 129.6 (2×C, C_{Arom}-C_{Arom}*), 132.4 (2×C, C_{Arom}-C_{Arom}*), 148.4 (2×C, C2-C2*), 151.5 (2×C, C15-C15*), 168.3 (2×C, C19-C19*), 171.13 (C, C16), 171.17 (C, C16*), 172.3 (2×C, C22-C22*)

HRMS (ESI): *m/z* calc. for $C_{22}H_{22}O_4N_3$ $[M-C_2O_2F_3]^+$ 392.1605, found 392.1609

$[\alpha]_D^{20}$ = - 1.15 (c = 0.54, MeOH)

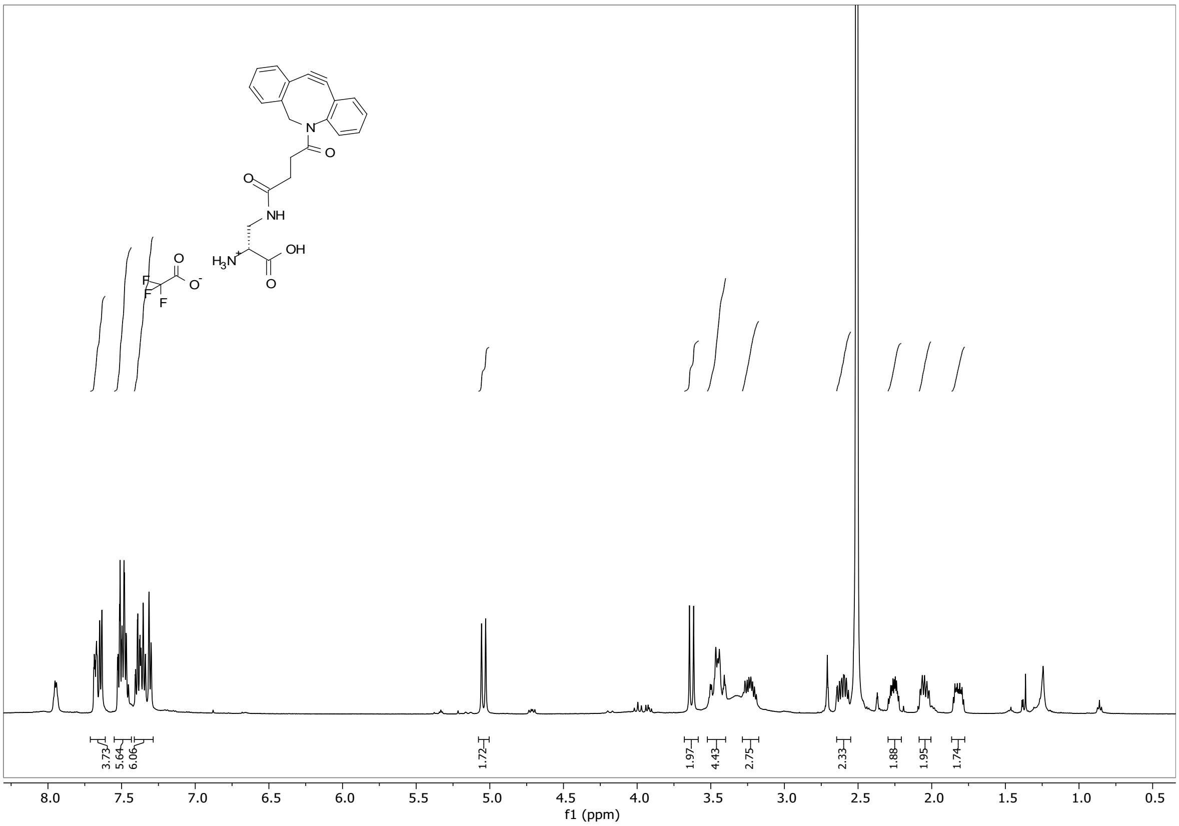
NH
OH
H_3N^+
O
F
O⁻
3.73
5.64
6.06
1.72
1.97
4.43
2.75
2.33
1.88
1.95
1.74
8.0 7.5 7.0 6.5 6.0 5.5 5.0 4.5 4.0 3.5 3.0 2.5 2.0 1.5 1.0 0.5
f1 (ppm)

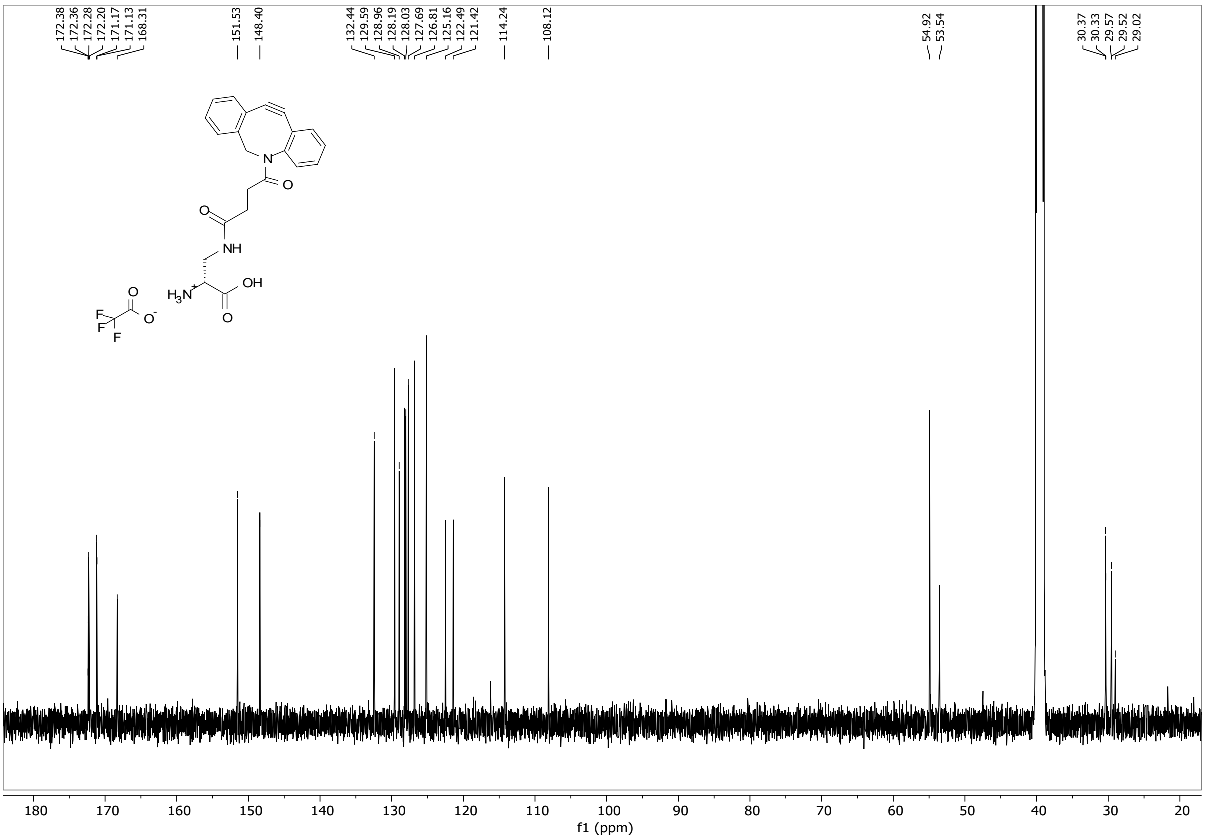
172.38
172.36
172.28
172.20
171.17
171.13
168.31
151.53
148.40
132.44
129.59
128.96
128.19
128.03
127.69
126.81
125.16
122.49
121.42
114.24
108.12
54.92
53.54
30.37
30.33
29.57
29.52
29.02
NH
OH
H_3N^+
O
F
O⁻
180 170 160 150 140 130 120 110 100 90 80 70 60 50 40 30 20
f1 (ppm)

3. PG labelling in *S. pneumoniae*

S. pneumonia R800 WT, R800 Δ*dacA* and D39 Δ*cps* strains were grown in brain heart infusion (BHI) at 37°C with 5% CO_2. The bacterial culture was diluted twice at 1:10 volume ratio in 10 mL of pre-warmed BHI to reach a steady-state growth at the exponential phase (OD_{600nm} ≈ 0.3). Exponential phase cultures were pelleted (9,000 x g, 5 min) and resuspended at a 6 x 10^8 cells/mL in pre-warmed BHI containing 250 µM D-Ala-cyclo-octynes and 40 µM azido-AF647 for 10 min at 37°C: concomitant labeling. Cells were then washed 3 times with PBS before immediate observation by conventional fluorescence microcopy.

For sequential labeling, bacteria were first incubated 10 min at 37°C in BHI containing 250 µM D-Ala-cyclo-octynes, then centrifuged and resuspended in BHI containing 40 µM azido-AF647. When specified, a pre-click was performed between 2.37 mM of D-Ala clyco-octynes and 382 µM of azido-AF647 fluorophore for the indicated time, before adding to bacteria for 10 min à 37°C (final concentration: 0.25 mM D-Ala-DIBO **8**, 10 µM azido-AF647).

Flow cytometry was performed on a MacsQuant VYB flow cytometer (Miltenyi) with a 561 nm laser and a 655/730 filter. Ten thousand events were analyzed for each run of analysis. Median fluorescence values of the cell population are shown.

For conventional fluorescence microscopy, labeled bacteria resuspended in PBS were deposited on a glass slide and immediately observed on an Olympus IX83 microscope with UPFLN 100 O-2PH/1.3 objective and a Hamamatsu Orca Flash4 CMOS camera. Images were acquired using the Cellsens V3 software (Olympus) and analyzed using FIJI software. Images from a same experiment were processed with the same brightness and contrast.

For dSTORM imaging, the bacterial pellet from a 10-mL culture in exponential phase was resuspended in 1/30th volume (about 300 µL) of pre-warmed BHI supplemented with the appropriate labeling probes. A mix of 3.8 mM D-Ala-DIBO **8** and 308 µM azido-fluorophore (azido-AF647 or azido-AF532) was prepared 90 min before its addition to the concentrated bacterial culture (final concentration: 0.25 mM D-Ala-DIBO **8**, 20 µM azido-fluorophore), together with 0.6 mM azido-Cho **2**. The sample was then incubated for 10 min at 37°C before being washed twice with PBS. Next, the cells were fixed with 2% paraformaldehyde (PFA) in PBS overnight at 4 °C. Cells were pelleted the next day (9,000 x g, 5 min) and resuspended into 160 µL PBS containing 30 µM DBCO-fluorophore (DBCO-AF532 or DBCO-AF647, avoiding the dye already used in the mix with D-Ala-DIBO). The samples were incubated at room temperature for 1 h, then washed 3 times with 1 mL PBS before microscopy observation.

Labelled cells were resuspended in dSTORM buffer containing 100 mM β-mercaptoethylamine and an oxygen-depleting system consisting of a GLOX enzyme mix (40 µg/mL catalase, 0.5 mg/mL glucose oxidase) in 75 mM Tris-HCl pH 8.0, 25 mM NaCl, 10% glucose (w/v). The sample was mounted between a microscopy slide and a high-precision coverslip as described previously by *Trouve et al.*.[4] The edges of the coverslip were then sealed using colorless nail polish.

The sample was observed with an Abbelight SAFe 360 commercial STORM microscope, which uses ASTER technology that provides a homogeneous sample excitation. The field was excited with a 640-nm scanning laser at maximum intensity (800 W/cm^2 of average effective power density at the sample level), upon which AF647 dyes started going through cycles of fluorescent state and dark state. When most AF647 fluorophores had displayed this transition, a set of 15 000 consecutive frames were taken with 50-ms exposure time. A second fluorescence signal acquisition was then carried out for AF532 in the same manner.

The two 15,000-frame sets were processed with the FIJI plugin ThunderSTORM, which localized the center of a Gaussian function fitted to each individual fluorescence signal, returned a data table containing the localization coordinates of all labelled molecules and conducted drifts correction by cross-correlation for each set of acquisition. These drifts corrected data tables were loaded onto NEOAnalysis program, which then align the two sets of acquisition using the Channel Realignment X/Y feature. Finally, the aligned data were processed by ImageJ/FIJI plugin ThunderSTORM to reconstruct the two super-resolution images individually, which were combined into a single image stack with 2 channels.

4. SPAAC kinetics study

To better understand the labeling efficiency of different clickable D-amino acids observed by fluorescence microscopy after reaction with clickable fluorophores, we determined the click reaction rates in PBS. More specifically, we studied the reactions of D-Ala-BCN **7** (DA-BCN in the graphics), D-Ala-DIBO **8** (DA-DIBO) and D-Ala-DBCO **9** (DA-BCN) with azido-AF488 (z488).

The concentration of azido-AF488 was determined by its absorbance at 494 nm, using the extinction coefficient 71,000 $M^{-1}\cdot cm^{-1}$ (https://www.thermofisher.com/fr/fr/home/brands/molecular-probes/key-molecular-probes-products/alexa-fluor/alexa-fluor-dyes-across-the-spectrum.html).

The concentration of the D-Ala-BCN **7**, D-Ala-DIBO **8** and D-Ala-DBCO **9** stock solution was determined by titration with azido-AF488 after reaction was complete. The required incubation times were first determined by time course experiments.

Determination of [D-Ala-BCN 7]$_{stock}$

In 2-µL reaction volumes of PBS, 1 µL of azido-AF488 stock solution was mixed with varying volumes (0 to 0.6 µL) of D-Ala-BCN **7** stock solution. After 6 and 7 days at 37°C, the reactions were diluted to 125 µL, and 10 µL were analyzed by electrophoresis in 17.5% acrylamide gel (29:1 acrylamide:bis-acrylamide) in the presence of 0.1% SDS with a Tris-Tricine buffer system after addition of 2 µL of loading buffer (1% SDS).

The absorbance at 494 nm of the diluted reactions was determined from absorbance spectra.

The relative amounts of the reacted (adduct) and unreacted azido-AF488 were determined from the non-saturated image of the gel trans-illuminated by near UV light (Geldoc BioRad) using ImageJ.

Volume of DA-BCN per 2 µL reaction

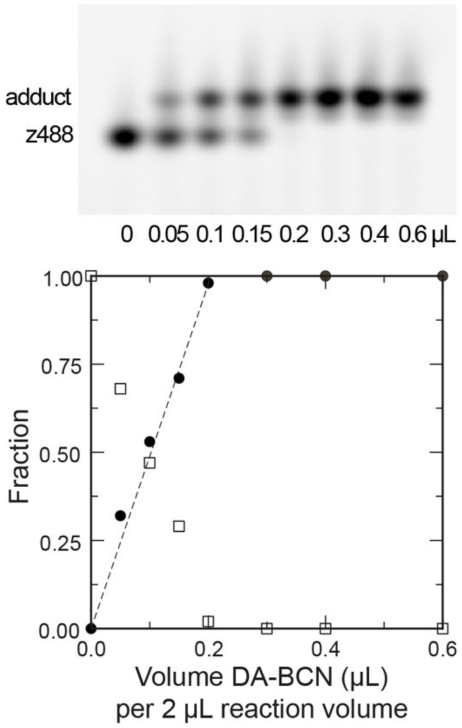

Abs$_{494nm}$ ± sd = 0.194 ± 0.025

$[\text{azido-AF488}]_{stock} = Abs_{494nm} \times 125 / 71000 = 340 \pm 40\ \mu M$

Titration linear fit = $5.0 \pm 0.2\ \mu L^{-1}$

VolumeEq = 1 / 5 = $0.20 \pm 0.01\ \mu L$

$[\text{D-Ala-BCN } \mathbf{7}]_{stock} = 0.340/0.20 = 1.7 \pm 0.3$ mM

To measure the rate of the reaction between D-Ala-BCN **7** and azido-AF488, the two compounds were mixed, and the reaction was stopped after various time intervals by the addition of a large excess of azido-choline that reacts rapidly with the remaining D-Ala-BCN **7**.

In PBS at 37°C, 20 µL of azido-AF488 at 1.4 µM were added to 20 µL of D-Ala-BCN **7** at concentrations of 0.17 (A), 0.34 (B), 0.68 (C) and 1.36 mM (D). At various times, 5 µL were withdrawn and added to 5 µL of azido-choline **2** at 100 mM. After addition of 2 µL loading buffer (1% SDS), samples were analyzed by electrophoresis as above.

The relative amounts of the reacted (adduct) and unreacted azido-AF488 were determined as above.

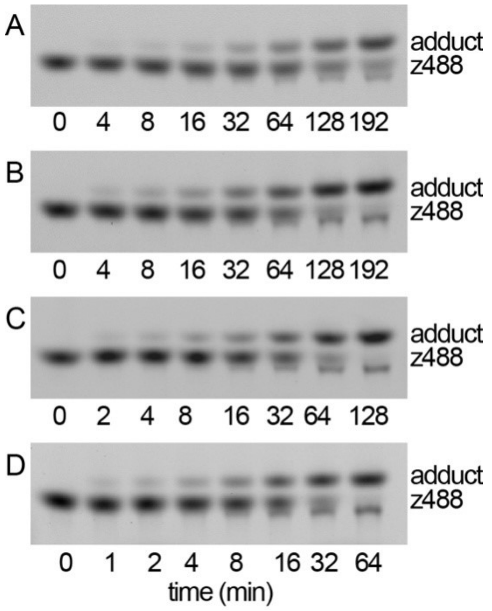

Apparent first order rate constants were obtained by fitting the band intensities to exponential curves. The second order rate constant was obtained by linear fitting of the apparent rates as a function of [D-Ala-BCN **7**]. Alternatively, the second order rate constant was obtained by global fitting of the time course data using the software Dynafit (Biokine).

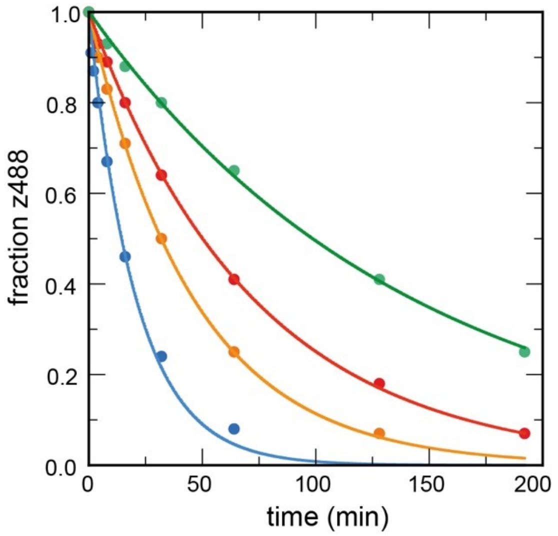

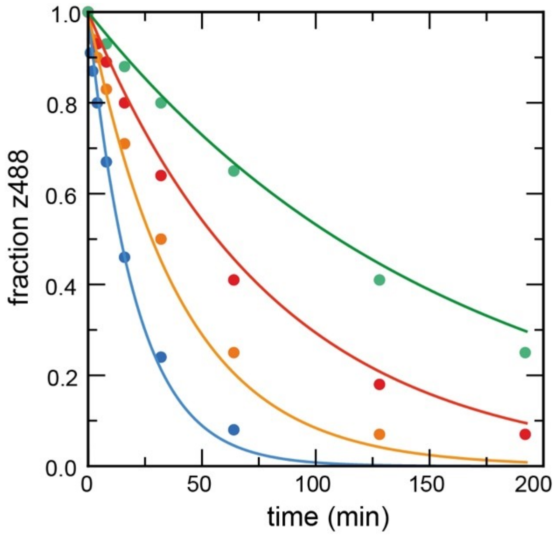

[D-Ala-BCN **7**] (µM)	k_{app} (min^{-1})
85±15	0.007
170±30	0.014
340±60	0.022
680±120	0.048

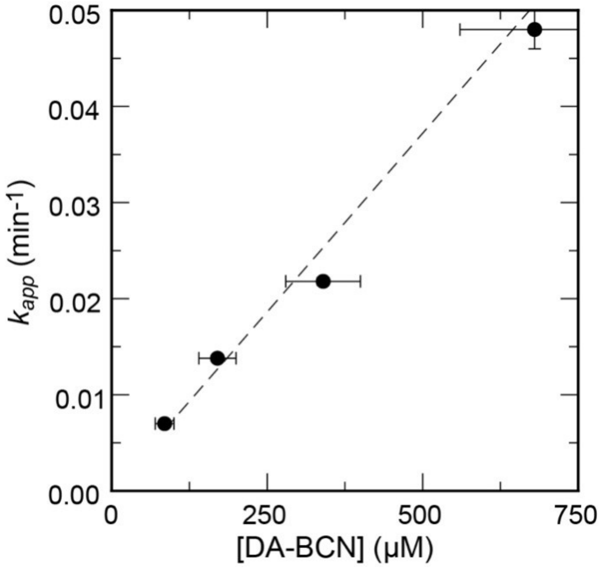

k = 75±4 (M^{-1}min^{-1}) = 1.25±0.07 (M^{-1}s^{-1}) error on fit

k = 74±2 (M^{-1}min^{-1}) = 1.23±0.04 (M^{-1}s^{-1}) error on fit (global fit)

k = 74±14 (M^{-1}min^{-1}) = 1.2±0.2 (M^{-1}s^{-1}) including error on concentration

Determination of [D-Ala-DIBO 8]$_{stock}$

In 2-µL reaction volumes of PBS, 1 µL of azido-AF488 stock solution was mixed with varying volumes (0 to 0.6 µL) of D-Ala-DIBO **8** stock solution. After 1 and 2 days at 37°C, the reactions were diluted to 125 µL, and 10 µL were analyzed by electrophoresis in 17.5% acrylamide gel (29:1 acrylamide:bis-acrylamide) without SDS with a Tris-Tricine buffer system after addition of 2 µL of loading buffer (without SDS).

The absorbance at 494 nm of the diluted reactions was determined from absorbance spectra.

The relative amounts of the reacted (adduct) and unreacted azido-AF488 were determined from the non-saturated image of the gel trans-illuminated by near UV light (Geldoc BioRad) using ImageJ.

Volume of DA-DIBO per 2 µL reaction

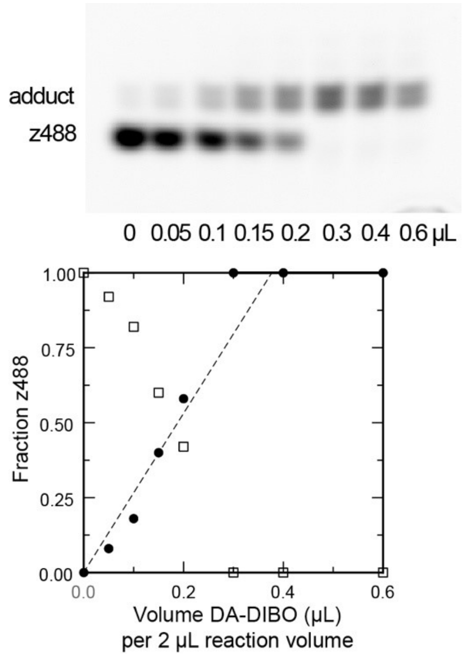

Abs_{494nm} ± sd = 0.20 ± 0.03

[azido-AF488]$_{stock}$ = Abs_{494nm} x 125 / 71000 = 350 ± 50 µM

Titration linear fit = 2.8 ± 0.2 µL^{-1}

VolumeEq = 1 / 2.8 = 0.26 ± 0.02 µL

[D-Ala-DIBO **8**]$_{stock}$ = 0.350/0.26 = 1.3 ± 0.3 mM

To measure the rate of the reaction between D-Ala-DIBO **8** and azido-AF488, the two compounds were mixed, and the reaction was stopped after various time intervals by the addition of a large excess of azido-choline that reacts rapidly with the remaining D-Ala-DIBO **8**.

In PBS at 37°C, 20 µL of azido-AF488 at 1.4 µM were added to 20 µL of D-Ala-DIBO **8** at concentrations of 33 (A), 65 (B), 130 (C) and 260 µM (D). At various times, 5 µL were withdrawn and added to 5 µL of azido-choline **2** at 100 mM. After addition of 2 µL loading buffer (no SDS), samples were analyzed by electrophoresis as above.

The relative amounts of the D-Ala-DIBO **8** reacted with azido-AF488 (adduct) and remaining azido-AF488 were determined as above.

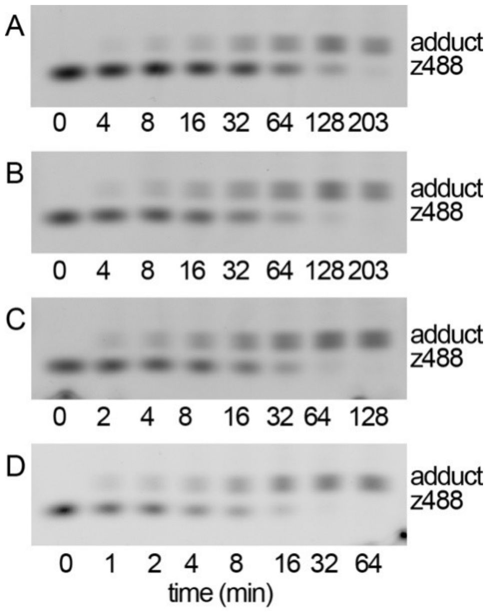

Apparent first order rate constants were obtained by fitting the band intensities to exponential curves. The second order rate constant was obtained by linear fitting of the apparent rates as a function of [D-Ala-DIBO **8**]. Alternatively, the second order rate constant was obtained by global fitting of the time course data using the software Dynafit (Biokine).

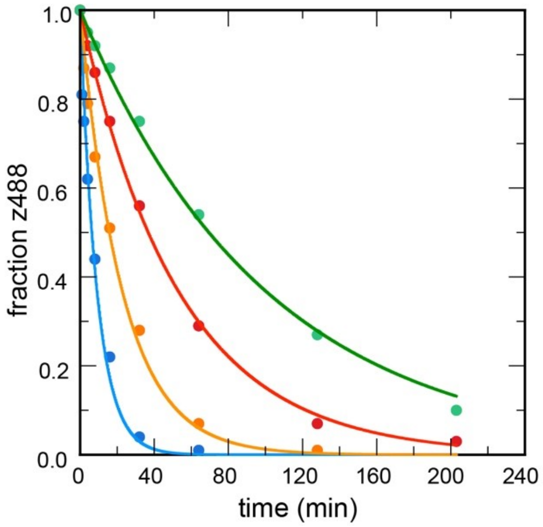

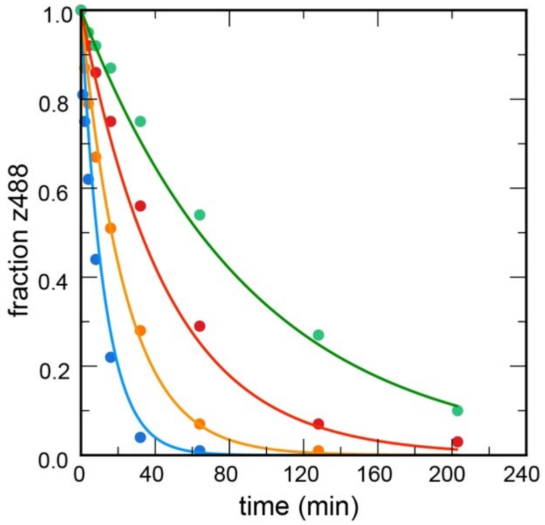

[D-Ala-DIBO **8**] (µM)	k_{app} (min^{-1})
16.3±3.6	0.010
32.5±7.5	0.019
65±15	0.044
130±30	0.110

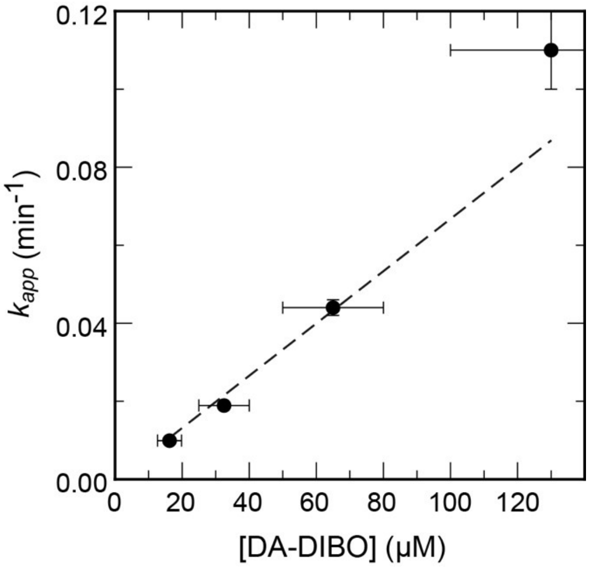

k = 670±60 (M^{-1}min^{-1}) = 11±1 (M^{-1}s^{-1}) error on fit

k = 670±20 (M^{-1}min^{-1}) = 11±0.3 (M^{-1}s^{-1}) error on fit (global fit)

k = 670±150 (M^{-1}min^{-1}) = 11±3 (M^{-1}s^{-1}) including error on concentration

Determination of [D-Ala-DBCO 9]$_{stock}$

In 4-µL reaction volumes of PBS, 2 µL of azido-AF488 stock solution was mixed with varying volumes (0 to 2 µL) of D-Ala-DBCO **9** stock solution. After 1 and 2 days at 37°C, the reactions were diluted to 125 µL, and 10 µL were analyzed by electrophoresis in 17.5% acrylamide gel (29:1 acrylamide:bis-acrylamide) without SDS with a Tris-Tricine buffer system after addition of 2 µL of loading buffer (without SDS).

The absorbance at 494 nm of the diluted reactions was determined from absorbance spectra.

The relative amounts of the reacted (adduct) and unreacted z488 were determined from the non-saturated image of the gel trans-illuminated by near UV light (Geldoc BioRad) using ImageJ.

Volume of DA-DBCO per 2 µL reaction

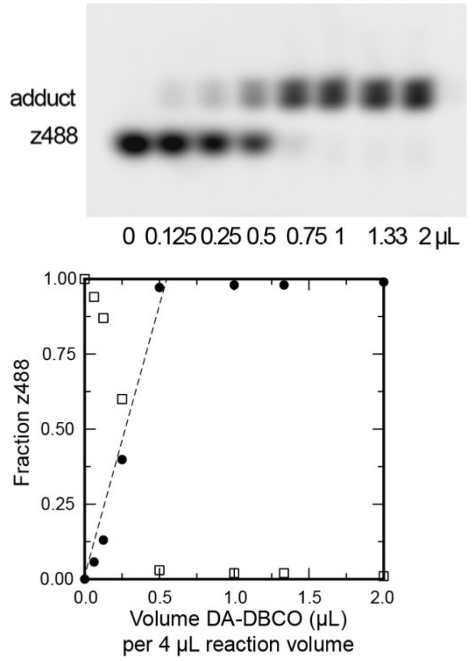

Abs$_{494nm}$ ± sd = 0.15±0.01

[azido-AF488]$_{stock}$ = Abs$_{494nm}$ x 62.5 / 71000 = 130±9 µM

Titration linear fit = 1.8±0.1 µL^{-1}

VolumeEq = 1 /1.8 = 0.55±0.03 µL

[D-Ala-DBCO **9**]$_{stock}$ = 2 x 0.130/0.55 = 470±60 µM

To measure the rate of the reaction between D-Ala-DBCO **9** and azido-AF488, the two compounds were mixed, and the reaction was stopped after various time intervals by the addition of a large excess of azido-choline that reacts rapidly with the remaining D-Ala-DBCO **9**.

In PBS at 37°C, 20 µL of azido-AF488 at 1.4 µM were added to 20 µL of D-Ala-DBCO **9** at concentrations of 19.8 (A), 39.5 (B) and 79 µM (C). At various times, 5 µL were withdrawn and added to 5 µL of azido-choline **2** at 100 mM. After addition of 2 µL loading buffer (no SDS), samples were analyzed by electrophoresis as above.

The relative amounts of the D-Ala-DBCO **9** reacted with azido-AF488 (adduct) and remaining azido-AF488 were determined as above.

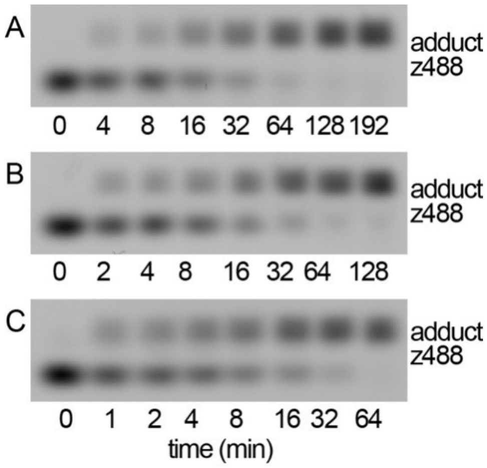

Apparent first order rate constants were obtained by fitting the band intensities to exponential curves. The second order rate constant was obtained by linear fitting of the apparent rates as a function of [D-Ala-DBCO **9**]. Alternatively, the second order rate constant was obtained by global fitting of the time course data using the software Dynafit (Biokine).

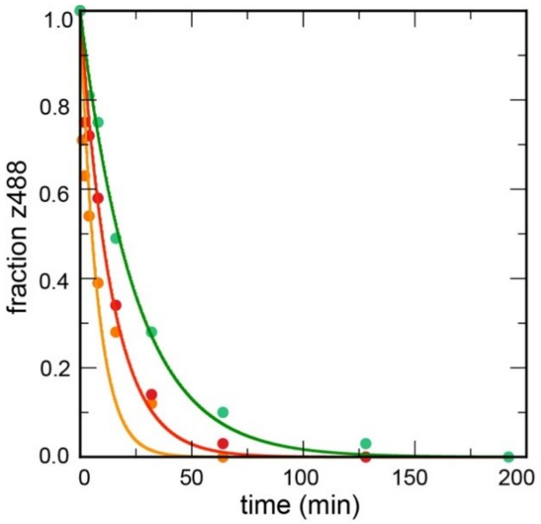

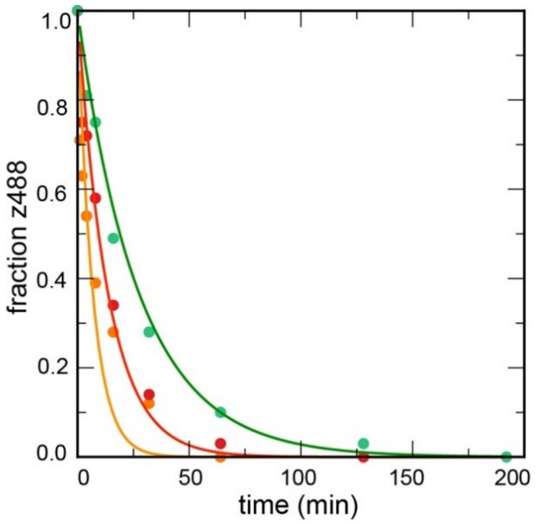

[D-Ala-DBCO **9**] (µM)	k_{app} (min^{-1})
9.9±0.7	0.041±0.002
19.8±1.4	0.071±0.06
39.5±2.8	0.13±0.02

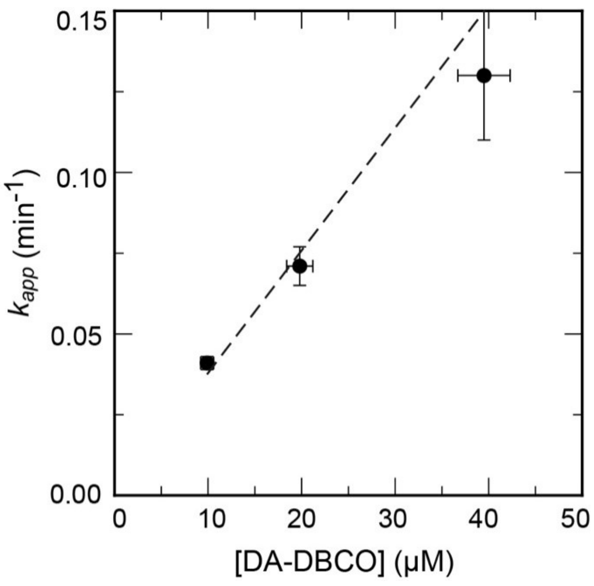

k = 3800±200 (M^{-1}min^{-1}) = 63±3 (M^{-1}s^{-1}) error on fit

k = 3800±300 (M^{-1}min^{-1}) = 63±5 (M^{-1}s^{-1}) error on fit (global fit)

k = 3800±500 (M^{-1}min^{-1}) = 63±8 (M^{-1}s^{-1}) including error on concentration

Summary Table

Reaction rate with azido-AF488 in PBS at 37°C	($M^{-1}s^{-1}$)
D-Ala-BCN **7**	1.2±0.2
D-Ala-DIBO **8**	11±3
D-Ala-DBCO **9**	63±8

5. References

(1) Ning, X.; Guo, J.; Wolfert, M. A.; Boons, G.-J. Visualizing Metabolically-Labeled Glycoconjugates of Living Cells by Copper-Free and Fast Huisgen Cycloadditions. *Angew. Chem. Int. Ed.* **2008**, *47*, 2253-2255. https://doi.org/10.1002/anis.200705456.

(2) Shieh, P.; Siegrist, M. S.; Cullen, A. J.; Bertozzi, C. R. Imaging Bacterial Peptidoglycan with Near-Infrared Fluorogenic Azide Probes. *Proc. Natl. Acad. Sci.* **2014**, *111*, 5456–5461. https://doi.org/10.1073/pnas.1322727111.

(3) Liu, C.; Li, T.; Rosi, N. L. Strain-Promoted "Click" Modification of a Mesoporous Metal–Organic Framework. *J. Am. Chem. Soc.* **2012**, *134*, 18886–18888. https://doi.org/10.1021/ja307713q.

(4) Trouve, J.; Zapun, A.; Arthaud, C.; Durmort, C.; Di Guilmi, A. M.; Söderström, B.; Pelletier, A.; Grangeasse, C.; Bourgeois, D.; Wong, Y. S.; Morlot, C. Nanoscale dynamics of peptidoglycan assembly during the cell cycle of Streptococcus pneumoniae. *Curr. Biol.* **2021,** *31*, 2844–2856.e6. https://doi.org/10.1016/j.cub.2021.04.041.

6. Appendix

Untreated images of the in-gel fluorescence used for the cyclooctyne titration and SPAAC kinetics determination. The original .tif files are available at https://doi.org/10.6084/m9.figshare.26310523 https://figshare.com/s/779df9bfd3d5add5418b

D-Ala BCN titration

Volume of DA-BCN **7** per 2 µL reaction from left to right: 0, 0.05, 0.1, 0.15, 0.2, 0.3, 0.4, 0.6 µL.

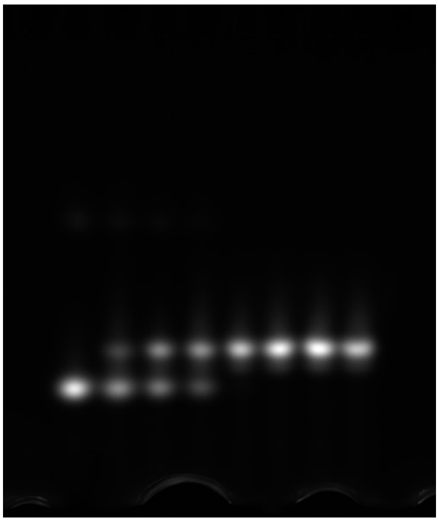

D-Ala BCN kinetics

20 µL of azido-AF488 at 1.4 µM were added to 20 µL of D-Ala-BCN **7** at concentrations of 0.17 (A), 0.34 (B), 0.68 (C) and 1.36 mM (D). At various times, 5 µL were withdrawn and added to 5 µL of azido-choline **2** at 100 mM.

A

From left to right, withdrawal times were 0, 4, 8, 16, 32, 64, 128, 192 min

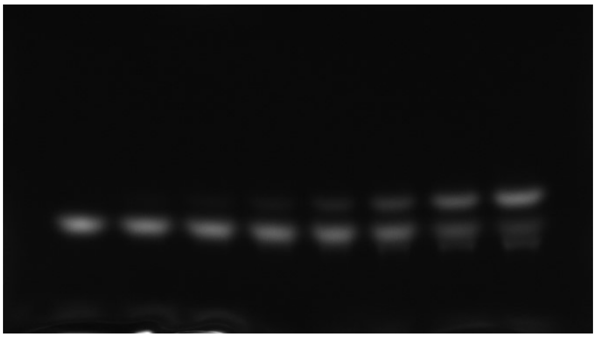

B

From left to right, withdrawal times were 0, 4, 8, 16, 32, 64, 128, 192 min

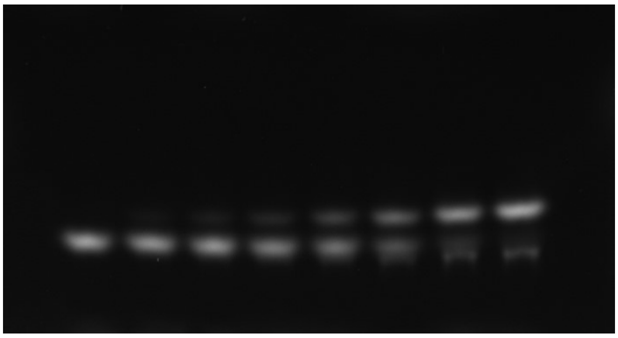

C

From left to right, withdrawal times were 0, 2, 4, 8, 16, 32, 64, 128 min

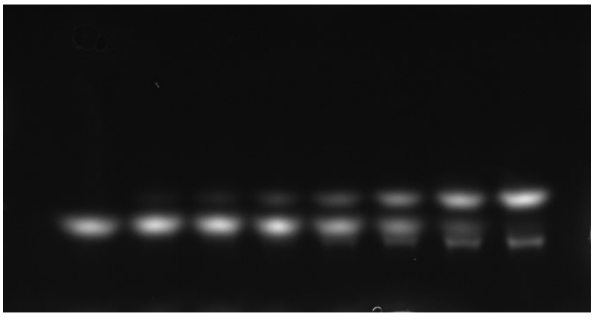

D

From left to right, withdrawal times were 0, 1, 2, 4, 8, 16, 32, 64 min

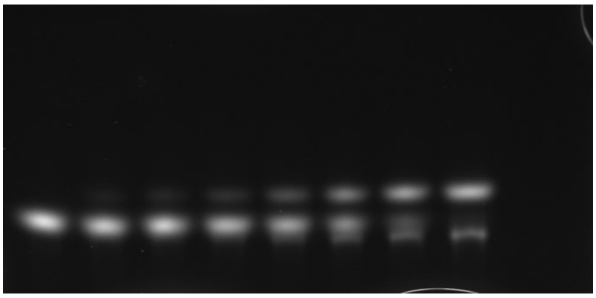

D-Ala DIBO titration

Volume of DA-DIBO per 2 µL reaction from left to right: 0, 0.05, 0.1, 0.15, 0.2, 0.3, 0.4, 0.6 µL.

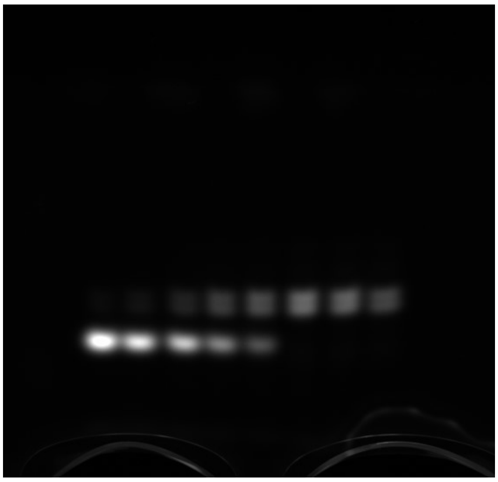

D-Ala DIBO kinetics

20 µL of azido-AF488 at 1.4 µM were added to 20 µL of D-Ala-DIBO **8** at concentrations of 33 (A), 65 (B), 130 (C) and 260 µM (D). At various times, 5 µL were withdrawn and added to 5 µL of azido-choline **2** at 100 mM.

A

From left to right, withdrawal times were 0, 4, 8, 16, 32, 64, 128, 203 min

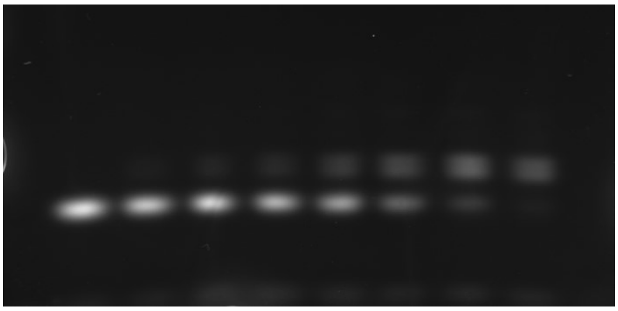

B

From left to right, withdrawal times were 0, 4, 8, 16, 32, 64, 128, 203 min

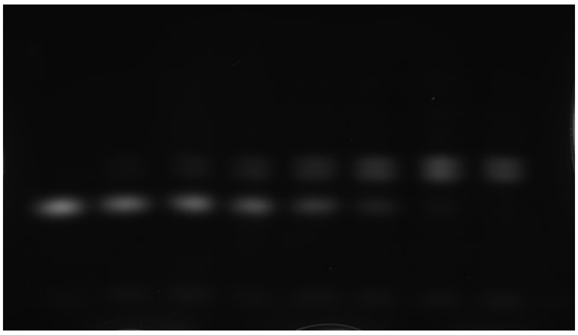

C

From left to right, withdrawal times were 0, 2, 4, 8, 16, 32, 64, 128 min

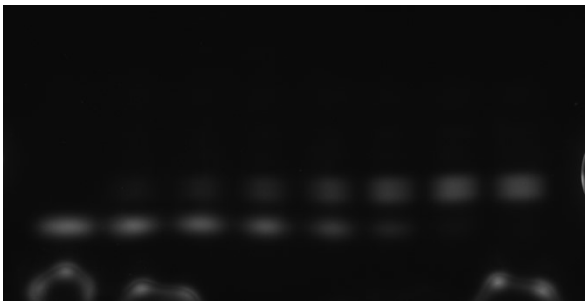

D

From left to right, withdrawal times were 0, 1, 2, 4, 8, 16, 32, 64 min

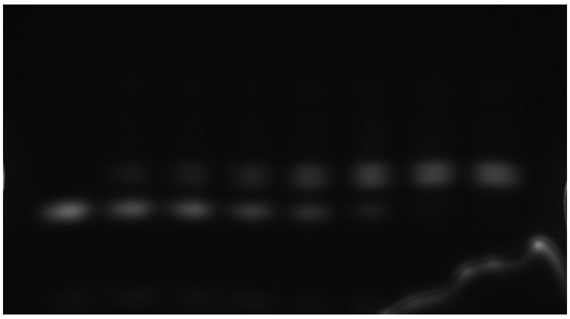

D-Ala DBCO titration

Volume of DA-DBCO per 4 µL reaction from left to right: 0, 0.125, 0.25, 0.5, 0.75, 1, 1.33, 2 µL.

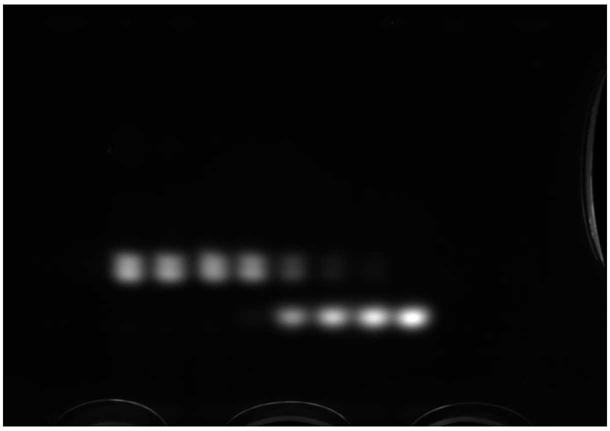

D-Ala DBCO kinetics

20 µL of azido-AF488 at 1.4 µM were added to 20 µL of D-Ala-DBCO **9** at concentrations of 19.8 (A), 39.5 (B) and 79 µM (C). At various times, 5 µL were withdrawn and added to 5 µL of azido-choline **2** at 100 mM.

A

From left to right, withdrawal times were 0, 4, 8, 16, 32, 64, 128, 192 min

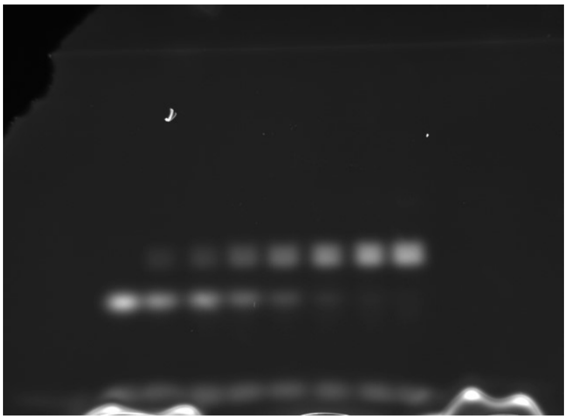

B

From left to right, withdrawal times were 0, 2, 4, 8, 16, 32, 64, 128 min

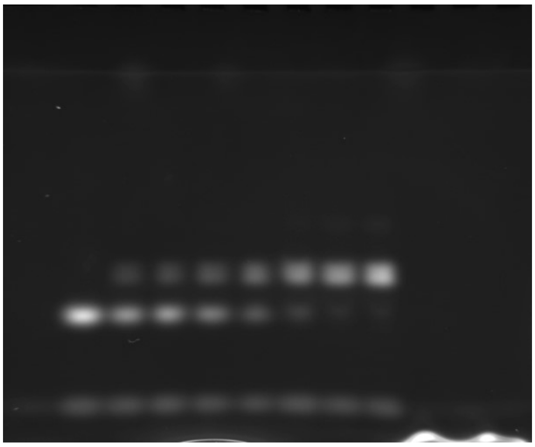

C

From left to right, withdrawal times were 0, 1, 2, 4, 8, 16, 32, 64 min

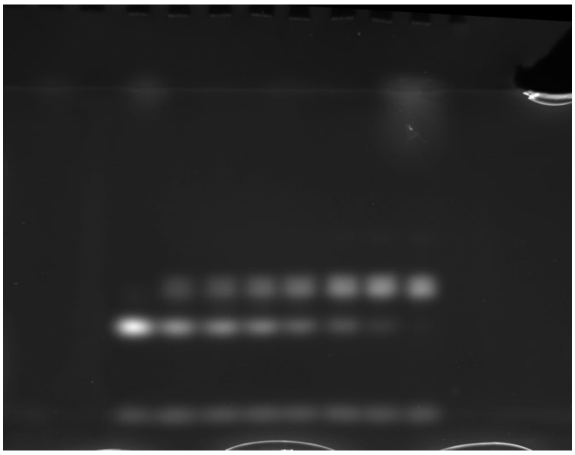

Supplement: RA-014-D4RA04945D-s001 [file RA-014-D4RA04945D-s001.pdf]
